# Citrus-leaf microemulsion controls postharvest mold through membrane disruption and host defense enhancement

**DOI:** 10.1016/j.fochx.2026.104048

**Published:** 2026-06-05

**Authors:** Lan-Tu Xiong, Jing Yi, Jiayu Xu, Gen Zhang, Yi-Lin Ma, Jing Jiao, Ri-Yuan Tang

**Affiliations:** a Key Laboratory of Advanced Materials for Facility Agriculture, Ministry of Agriculture, College of Materials and Chemical Engineering, South China Agricultural University, Guangzhou 510642, PR China; b State Key Laboratory of Green Pesticide, Integrative Microbiology Research Centre, Guangdong Provincial Key Laboratory of Microbial Signals and Disease Control, College of Plant Protection, South China Agricultural University, Guangzhou 510642, PR China; c The Second Clinical Medical College, Southern Medical University, Guangzhou 510515, PR China; d School of Chemistry and Civil Engineering, Shaoguan University, Shaoguan 512005, PR China

**Keywords:** Agro-waste valorization, Postharvest decay, Green chemistry, *Penicillium* decay, Microemulsion, Eco-friendly fungicide, Circular economy

## Abstract

Postharvest fungal decay causes substantial losses in the global citrus industry. To address fungicide resistance and waste management, this study developed a circular “from-tree-to-fruit” strategy, valorizing citrus leaf residues into a stable 7% microemulsion (7% ME). Optimized extraction yielded a Citrus Leaf Extract (CLE) enriched with the antifungal 2,4-di-tert-butylphenol (2,4-DTBP). At 5 × EC_50_, 7% ME controlled blue mold (*Penicillium italicum*) and green mold (*P. digitatum*) by 77.4% and 94.5%, respectively, compared with 71.0% and 81.3% for thiabendazole. Mechanistic analyses revealed a dual mode of action: direct disruption of fungal membrane integrity and induction of key host defense enzymes, including superoxide dismutase (SOD), peroxidase (POD), and catalase (CAT), thereby enhancing the fruit's innate resistance. Furthermore, ecotoxicological assessments confirmed the safety of the 7% ME for non-target organisms, including silkworms, zebrafish, and earthworms. This study presents a scalable, eco-friendly framework for transforming agricultural byproducts into high-performance biopesticides.

## Highlights

• Citrus leaves are valorized as a sustainable source of antifungal bioactives.
• 7% Microemulsion formulation significantly enhances the bioavailability.
• Achieves up to 94.5% control against *Penicillium* postharvest diseases.
• Acts *via* host defense induction and fungal membrane disruption.
• Safe to non-target organisms and supports sustainable postharvest use.

## Graphical abstract

**Figure:**
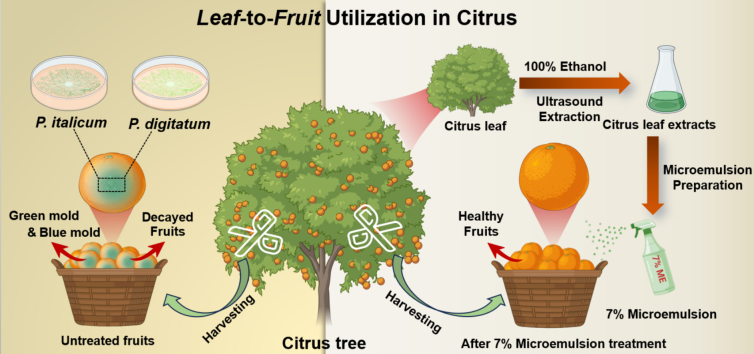

## 1 Introduction

Citrus fruits act as a cornerstone of global nutrition and agricultural trade, with an annual production exceeding 150 million metric tons (Han et al., 2024). However, the industry faces a persistent dual challenge: managing massive volumes of agro-industrial waste and preventing postharvest decay (Du et al., 2023; Lin et al., 2025). Fungal diseases are the main cause of losses after harvest. Among them, *Penicillium italicum* and *P. digitatum* are the most common pathogens. These fungi can cause more than 50% of fruit spoilage during storage and transport (Arruda et al., 2025; Peng et al., 2025). Synthetic fungicides such as imazalil and thiabendazole are still widely used. They show strong antifungal activity. However, their long-term use has led to resistant strains. In addition, strict limits on chemical residues have been introduced in many regions (Dong et al., 2025; Piancatelli et al., 2026; Zhang et al., 2023a). These pressures have accelerated the search for biodegradable alternatives that ensure environmental compatibility without long-term ecological accumulation (Zhao et al., 2025).

At the same time, citrus pruning produces a large amount of plant residues. Most of these materials are discarded as waste. From the perspective of food chemistry, these residues are valuable. They contain many secondary metabolites with biological activity. However, they are not fully utilized (Gómez-Mejía et al., 2019; Ligarda-Samanez et al., 2025; Qi et al., 2021). Unlike the well-characterized volatile essential oils, the non-volatile lipophilic fraction has received less attention. This fraction includes polymethoxylated flavones, terpenes, and lipophilic phenols. These compounds are closely related to plant defense against pathogens (Desmiaty et al., 2025; Huang et al., 2025). The full utilization of these ingredients is in line with the concept of circular bioeconomy, such as converting agricultural residues into high-value food preservatives (Arshad et al., 2022). This approach can be described as a “from tree to fruit” strategy. It uses the plant's own defense system to protect harvested fruits (Tian et al., 2024).

To explore this fraction, it is necessary to identify key active compounds. In this study, 2,4-di-tert-butylphenol (2,4-DTBP) was identified as a key antimicrobial marker, noted for its ability to disrupt fungal cell membranes and redox homeostasis (Fan et al., 2023). However, these lipophilic compounds have low water solubility. This property limits their practical use. As a result, their effectiveness is reduced. Microemulsion systems provide a possible solution to this problem by improving the dispersion of hydrophobic compounds (Almasi et al., 2021; Inan-Çinkir et al., 2024). By creating a thermodynamically stable and isotropic system, microemulsions can solubilize hydrophobic actives like 2,4-DTBP, facilitating their interaction with fungal targets such as sterol 14α-demethylase (CYP51) (Xiong et al., 2022). Therefore, two steps are important. One is the identification of active compounds. The other is the design of an effective delivery system. Together, they help improve the value of citrus leaf residues.

Consequently, this study aimed to develop a high-performance botanical fungicide by integrating chemical extraction optimization with advanced formulation science. Specifically, we utilized response surface methodology (RSM) to maximize the recovery of bioactive lipophiles from *Citrus reticulata* leaves and employed GC–MS and HPLC-MS to definitively characterize the natural occurrence of 2,4-DTBP. A stable microemulsion system was then engineered to overcome solubility limitations. The antifungal activity was tested *in vivo*. In addition, membrane permeability assays and molecular docking were used to explore the mechanism. This work provides a technical basis for transforming citrus processing waste into a functional botanical postharvest treatment.

## 2 Materials and methods

### 2.1 Pathogens, plant material, and chemicals

*P. italicum* (GDMCC 3.1010) and *P. digitatum* (GDMCC 3.1022) were from the College of Plant Protection, South China Agricultural University. Fresh citrus leaves (*Citrus reticulata* Blanco, cultivar ‘Ponkan’) were collected in February 2023 from Changpu Village, Lanshan County, Yongzhou City, Hunan Province, China (25°03′ N, 112°13′ E). Full experimental details and instrumentation are provided in the Supporting Information.

### 2.2 Optimization of CLE preparation

Fresh citrus leaves (*Citrus reticulata* Blanco) were washed with water and then divided into two groups. One group was air-dried at room temperature in a well-ventilated, shaded area protected from direct sunlight until constant weight, while the other was dried in an oven at 45 °C for 60 h. After drying, the leaves were ground into powder and passed through a 60-mesh sieve. For solvent screening, 1.0 g of the powder was mixed with 5 mL of different solvents, including anhydrous ethanol, petroleum ether, and ethyl acetate. Ultrasonic extraction was performed at 30 °C for 0.5 h. The extracts were then filtered through a 0.22 μm membrane. After filtration, the samples were concentrated under reduced pressure (−0.09 MPa, 45 °C) using a rotary evaporator.

Single-factor experiments were first carried out to evaluate the effect of ethanol concentration. Three levels were tested, including anhydrous ethanol, 75%, and 50%. Based on these results, the optimal ethanol concentration was selected and used in the following experiments. Further experiments were then conducted to examine ultrasonic temperature (20–80 °C), extraction time (0.5–4 h), and solvent-to-material ratio (1:5–1:25, m/v). These factors were adjusted to improve extraction efficiency. Finally, these parameters were optimized using response surface methodology. A Box–Behnken design was applied with Design-Expert 10.0.4 software. Ultrasonic temperature, extraction time, and solvent-to-material ratio were used as independent variables.

### 2.3 *In vitro* antifungal activity assay

The antifungal activity of the crude extracts obtained from different extraction conditions was assessed using a modified mycelial growth inhibition assay (Xiong et al., 2022). The dried extracts were first dissolved in dimethyl sulfoxide (DMSO) and then added to the PDA medium at the specified concentration. The final concentration of DMSO in the medium was strictly maintained at 0.5% (*v/v*). A solvent control containing 0.5% DMSO was concurrently evaluated, which confirmed the absence of any solvent-induced fungotoxicity. Aliquots (15 mL) of the amended media were dispensed into sterile 90-mm Petri dishes. Fungal inoculum (6-mm diameter agar plugs) was aseptically excised specifically from the expanding margins of 5-day-old actively growing cultures to ensure uniform mycelial age and density while avoiding heavily sporulated central regions. The uniform plugs were then transferred to the center of each plate. All plates were incubated at 28 ± 1 °C under dark conditions for 72 h. The percentage inhibition of radial growth was calculated using the formula:

$$\mathrm{Inhibition rate}\left(\%\right)=\left[\left(C–T\right)/\left(C–0.6\right)\right]\times 100$$

Where *C* and *T* are the average mycelial growth diameters (cm) in control and treatment plates, respectively, with the initial plug diameter (0.6 cm) subtracted to normalize growth. Three independent replicates were performed for each treatment.

### 2.4 Chemical composition analysis of CLE

The chemical composition of CLE was analyzed by gas chromatography–mass spectrometry (GC–MS) to profile volatile and semi-volatile constituents. Although CLE contained both volatile and non-volatile compounds, a large proportion of its bioactive constituents was extractable and detectable under the GC–MS analytical conditions (Castillo et al., 2013). The extract was dissolved in HPLC-grade acetone and filtered through a 0.22 μm organic membrane to obtain a 1000 mg/L test solution, as acetone ensured solubility of semi-volatile constituents. To avoid interference from antioxidants released by plastics, all experiments were carried out using pre-baked borosilicate glassware. Plastic materials were not used during the procedures. Solvent blanks were processed identically and showed no detectable 2,4-DTBP signal, indicating that 2,4-DTBP was not introduced from solvents or the analytical background under the applied conditions. Furthermore, the extraction and GC–MS analyses were performed on three independent biological batches of citrus leaves, and 2,4-DTBP was consistently detected across all batches, demonstrating its stable presence as an endogenous metabolite. GC–MS analysis was performed using a HP-5 quartz capillary column (30 m × 0.32 mm × 0.25 μm). The injector temperature was set at 280 °C. The oven was first held at 50 °C for 2 min. It was then increased to 280 °C at a rate of 10 °C/min and kept at this temperature for 5 min. Mass detection was performed using an electron impact (EI) ionization source at 70 eV. The ion source temperature was 230 °C, and the quadrupole temperature was set to 150 °C. A solvent delay time of 4 min was applied during analysis. Compounds were identified by comparing spectra with the NIST library, and most accepted matches had similarity indices above 90%. Compounds with lower similarity were regarded as tentative assignments. While CLE also contains non-volatile phytochemicals, the GC–MS approach selectively profiles volatile and semi-volatile fractions vaporized under the applied conditions, making it suitable for compositional screening.

The presence of 2,4-DTBP in CLE was further confirmed and validated using HPLC–MS analysis. Separation was performed on a C18 reverse-phase column (250 mm × 4.6 mm, 5 μm). Methanol was used as the mobile phase at a flow rate of 1.0 mL/min. The column temperature was maintained at 30 °C. The detection wavelength was 254 nm with an injection volume of 2 μL. Both the standard and extract solutions were prepared at 10 mg/L. Mass detection was carried out in negative electrospray ionization (ESI^−^) mode. The capillary voltage was −4500 V, and the desolvation temperature was 400 °C. The selected ion at *m*/*z* 205.2 ([M–H]^−^) was used for compound confirmation.

### 2.5 Development and optimization of a 7% ME for antifungal application

The microemulsion was prepared according to a previously reported emulsifiable oil method with minor modifications (Zhang et al., 2009). Solvent screening was first conducted by evaluating the solubility of CLE (10 g/L) and its low-temperature stability after storage at 4 °C for 72 h in different organic solvents. After ethanol was selected as the optimal solvent, the suitable HLB range was determined by assessing formulation clarity before and after water dilution across HLB values of 8–16. Binary surfactant systems were then screened by combining the anionic surfactant calcium dodecylbenzenesulfonate (CDBS) with different nonionic surfactants, including 1601#, 1602#, Tween-20 (T-20), and Tween-80 (T-80). Based on formulation stability and antifungal activity, the final optimized 7% ME consisted of 7% CLE, 27% anhydrous ethanol, 4% CDBS, 16% Tween-20, and 46% water. For preparation, CLE, ethanol, CDBS, and Tween-20 were mixed sequentially, followed by the slow addition of water under continuous stirring at 500 rpm until a transparent and homogeneous microemulsion system was formed.

The 7% ME underwent comprehensive evaluation including: (1) Physicochemical properties: the transparency range was determined by temperature cycling (−20 °C to 80 °C at 1 °C/min). The pH was measured in 1% aqueous dispersions according to GB/T 1601–1993. Microscopic observation was used to assess homogeneity; (2) Stability tests: stability was examined in standard hard water (342 mg/L, 30 ± 2 °C, GB/T 1603–2001). Cold storage was tested at 0 ± 2 °C for 7 d with intermittent mixing. Accelerated aging was carried out at 54 ± 2 °C for 14 d (GB/T 19136–2003). Centrifugation stability was evaluated at 500–600 ×g for 15 min; (3) Functional performance: foam properties were analyzed according to GB/T 28137–2011. Foam volume was required to remain below 25 mL after 1 min. Droplet size distribution was measured using dynamic light scattering.

To further evaluate the practical applicability of 7% ME as a sprayable stock formulation, additional formulation properties were assessed. The apparent viscosity of the undiluted 7% ME was measured at 25 ± 1 °C using a rotational viscometer and expressed as mPa·s. Dilution stability was evaluated by diluting the 7% ME stock formulation with standard hard water at ratios of 1:100, 1:200, and 1:500. The diluted working solutions were stored at 25 °C for 24 h and then visually examined for transparency, turbidity, visible particles, precipitation, and phase separation before and after gentle shaking. Sprayability was assessed by adding each diluted working solution into a handheld sprayer and continuously spraying 50 times, during which nozzle clogging, spray continuity, and visible pformation were recorded. Short-term light stability was evaluated by exposing the 7% ME to continuous illumination at 4000–6000 lx in a light incubator for 24 h. A dark control was prepared by wrapping the same formulation with aluminum foil and incubating it under the same temperature conditions. After exposure, the samples were visually examined for transparency, turbidity, visible particles, precipitation, and phase separation. All experiments were performed in triplicate with appropriate controls.

### 2.6 Biocontrol efficacies of 7% ME against green mold and blue mold

The antifungal efficacy of different formulations against citrus green mold (*P. digitatum*) and blue mold (*P. italicum*) was evaluated following the method of (Zhang et al., 2023a) with minor modifications. Uniformly sized, commercially mature mandarin oranges (*Citrus reticulata*) free of visible defects were surface-disinfected by immersion in 2% (*v*/v) sodium hypochlorite (NaClO) for 2 min, followed by triple-rinsing with distilled water and air-dried at room temperature. The experiment was arranged in a completely randomized design. Each treatment consisted of three biological replicates, with 20 fruits per replicate (total *n* = 60 fruits per treatment). Two artificial wounds (2 × 2 mm) were created at equatorial positions using sterile bamboo picks. Test formulations (SterileddH_2_O, CLE, 7% ME, and TBZ) were applied to wound sites at a concentration of 5× half-maximal effective concentration (EC_50_) for each respective pathogen (*P. digitatum* or *P. italicum*) (*e.g.*, the absolute application concentrations of the positive control TBZ were 0.40 mg/L against *P. digitatum*) by spraying onto the wound sites (approx. 2 mL per wound) using a handheld sprayer until runoff, a standard method deliberately chosen to ensure uniform tissue saturation and to simulate commercial packinghouse application practices. This concentration was selected based on prior *in vitro* dose–response data showing consistent and near-maximal inhibition, allowing for a standardized, potency-normalized comparison of treatment efficacy under postharvest conditions. After air-drying, 10 μL of *P. digitatum* or *P. italicum* conidial suspensions (10^6^ CFU/mL) were carefully pipetted onto each wound site. Fruits were incubated in humidity-controlled chambers (25 ± 1 °C, 80 ± 5% RH) for 5 days. Disease severity was quantified using a 6-point scale: 0 = no infection; 1 = ≤ 5% lesion area; 2 = 5–15%; 3 = 15–30%; 4 = 30–50%; 5 ≥ 50% infection. The disease index (DI) and relative control efficacy (RE) were calculated as:

$$\mathrm{DI}=\left[\Sigma \;\left(\mathrm{severity grade}\times \mathrm{infected fruits}\right)/\left(\mathrm{total fruits}\times 5\right)\right]\times 100$$

$$\mathrm{RE}=\left[\left({\mathrm{DI}}_{\mathrm{control}}–{\mathrm{DI}}_{\mathrm{treatment}}\right)/{\mathrm{DI}}_{\mathrm{control}}\right]\times 100.$$

### 2.7 Defense related enzymes detection

According to a previous study, citrus fruits at commercial maturity were used to evaluate the induction of defense-related enzymes (Tian et al., 2023). Briefly, uniform wounds were created on the fruit surface, and fruits were treated with 7% ME at 5 × EC_50_ by spraying (approx. 2 mL per fruit) until runoff to ensure the surface was uniformly wet, while control fruits were treated with sterile distilled water. After air-drying, wounds were inoculated with 10 μL aliquots of *P. digitatum* or *P. italicum* spore suspensions (10^6^ CFU/mL). All fruits were incubated at 25 °C and 85 ± 5% relative humidity in a controlled chamber. Pericarp tissues surrounding the wound sites (specifically, a cylindrical tissue block of 15 mm in diameter centered on the wound, encompassing the entire flavedo and albedo layers to a depth of approximately 2–3 mm, strictly excluding the underlying pulp) were collected from three randomly selected fruits per treatment at each time point and immediately frozen in liquid nitrogen for subsequent analysis. Activities of SOD were determined using assay kits (Beijing Solarbio Science & Technology Co., Ltd.), while POD and CAT activities were measured using kits from Nanjing Jiancheng Bioengineering Institute. All assays were performed according to the manufacturers' instructions.

### 2.8 Morphological alterations in *P. italicum* and *P. digitatum* hyphae induced by 7% ME

Hyphal morphological changes induced by the 7% ME were assessed using a previously described method (Zhang et al., 2019). To better understand the mode of antifungal action of 7% ME, the hyphal morphology of *P. italicum* and *P. digitatum* treated with 7% ME was observed by scanning electron microscopy (SEM). For SEM analysis, 7% ME was incorporated into PDA to prepare culture media supplemented with 7% ME at a concentration of 1 × EC_50_ for *P. italicum* and *P. digitatum*, respectively. PDA plates without the addition of 7% ME served as the control. A 6-mm mycelial disc of *P. italicum* and *P. digitatum* was then placed at the center of each medium, and all plates were incubated at 28 °C in the dark for 72 h. Following incubation, mycelial fragments (10 mm × 6 mm × 2 mm) were excised from the colony margins. The samples were fixed with 2.5% glutaraldehyde at 4 °C, rinsed three times with 0.1 M phosphate-buffered saline (PBS), and post-fixed with 1% (*w*/*v*) osmium tetroxide solution. After critical-point drying and gold sputter-coating, the treated mycelia were examined using an XL-30-ESEM scanning electron microscope (FEI, Eindhoven, Netherlands).

### 2.9 Determination of cell membrane permeability

Cell membrane relative permeability in *P. italicum* and *P. digitatum* was measured following treatment with 7% ME, using a previously described method (Xiong et al., 2022). Mycelial discs (6 mm) were precultured in potato dextrose broth (PDB, 200 mL) at 28 °C and 160 rpm for 5 days. The mycelia were then harvested by filtration, washed three times with sterile phosphate-buffered saline (PBS), and resuspended in PBS to prepare a standardized suspension. Aliquots equivalent to 1 g (wet weight) of *P. italicum* or *P. digitatum* mycelia were treated with 7% ME at concentrations of 1×, 2×, and 4 × EC_50_, respectively. Electrolyte leakage was monitored by measuring conductivity at 0 (*T*_*0*_), 0.5, 1.5, 3, 6, 12, 24, and 48 h after treatment (*T*_*1*_). Total conductivity (*T*_*2*_) was determined after boiling and cooling the samples. Relative membrane permeability was calculated as:

$$\mathrm{Relative electric conductivity}\left(\%\right)=\left[\left(T_{1}–T_{0}\right)/\left(T_{2}–T_{0}\right)\right]\times 100$$

### 2.10 Determination of cytoplasmic content leakage

Following the pretreatment procedure (Zhang et al., 2019), mycelia of *P. italicum* and *P. digitatum* (0.5 g fresh weight) were collected, washed twice with sterile distilled water, and resuspended in 0.01 M phosphate-buffered saline (PBS, pH 7.0). The mycelial suspensions of *P. italicum* and *P. digitatum* were exposed to 7% ME at 1×, 2×, and 4 × EC_50_ values specific to each fungus, followed by incubation at 28 °C for 6 h to assess physiological and membrane-damaging effects. This specific 6-h time point was selected based on established literature protocols (Zhang et al., 2019) to capture the acute phase of initial macromolecular leakage while effectively minimizing the potential secondary degradation of released nucleic acids and proteins by extracellular enzymes during prolonged incubation. After centrifugation (4000 ×*g*, 10 min), nucleic acid leakage in the supernatant was quantified by measuring absorbance at 260 nm (OD_260_) for nucleic acids and at 280 nm (OD_280_) for proteins using a UV–Vis spectrophotometer (UV1902PC, Shanghai Instrument Co.). Three biological replicates were performed for each treatment, including blank controls containing PBS only.

### 2.11 RNA extraction and quantitative real-time PCR (qRT-PCR) analysis

To examine whether 7% ME affected ergosterol biosynthesis-related responses, the transcriptional profiles of *CYP51A*, *CYP51B*, *CYP51C*, and *ERG6* in *P. italicum* and *P. digitatum* were evaluated. The mycelial suspensions were prepared as described in section 2.10 and treated with the 7% ME at their respective EC_50_ concentrations for 6 h. To eliminate potential transcriptional background noise caused by the formulation excipients, a blank microemulsion (Blank ME, comprising 27% anhydrous ethanol, 4% CDBS, 16% Tween-20, and 53% sterile water) was diluted by the exact same dilution factor and served as the vehicle control. Following the 6 h incubation, mycelia were harvested, rapidly frozen in liquid nitrogen, and ground into fine powder. Total RNA was extracted using the Fungal RNA Extraction Kit (Vazyme Biotech Co., Ltd., Guangzhou, China) according to the manufacturer's protocol. The concentration and purity of the extracted RNA were assessed using a NanoDrop spectrophotometer. Subsequently, cDNA was synthesized using a reverse transcription kit (Vazyme Biotech Co., Ltd., Guangzhou, China). The qRT-PCR assays were performed on a real-time PCR system using SYBR Green Master Mix. The *Actin* gene of each respective species was utilized as the endogenous reference gene to normalize the expression levels. The relative gene expression was calculated using the classic 2^−ΔΔCt^ method, with the expression level of the Blank ME control group designated as 1. The specific primer sequences used for the amplification are listed in Table S20 and S21. For each treatment, three independent biological replicates were prepared, and each biological replicate was analyzed with three technical replicates in the qRT-PCR assay.

### 2.12 Molecular docking

Based on physiological evidence demonstrating cell membrane disruption, the active compound 2,4-DTBP was selected for molecular docking studies against the target proteins. Specifically, the well-characterized CYP51A isoform (historically referred to as simply CYP51 prior to the discovery of its paralogs) was selected for both *P. digitatum* (UniProt ID: A1XG20) and *P. italicum* (UniProt ID: Q12664). The three-dimensional structures of these CYP51A proteins were predicted using AlphaFold. This approach provides reliable structural models for docking analysis. Docking simulations were carried out using AutoDock Vina. The ligand 2,4-DTBP (PubChem CID: 7311) was prepared in advance and optimized before docking. Protein structures were first processed before docking. Water molecules were removed, and polar hydrogen atoms were added. A grid box was then defined to cover the active site of each protein. Docking results were analyzed based on binding affinities and interaction profiles, allowing for the identification of potential binding modes between the ligand and the target proteins (Guo et al., 2025).

### 2.13 Toxicological evaluation methodology for 7% ME in non-target organisms: Silkworm, zebrafish, and earthworm

The silkworm (*B. mori*), zebrafish (*D. rerio*), and earthworm (*E. fetida*) were selected as test organisms. These species represent terrestrial, aquatic, and soil environments, respectively. They were used to assess the potential environmental toxicity of the formulation. All organisms were obtained from the College of Plant Protection, South China Agricultural University (Guangzhou, China). Before the experiment, they were acclimated for 7 days.

#### 2.13.1 Silkworm bioassay

The leaf-dip method was employed to assess the biological activity of the 7% ME derived from citrus extract. Four concentrations were prepared, including 200, 100, 50, and 25 mg/L. Each solution was prepared by dissolving 7% ME in 20 mL of deionized water. Fresh mulberry leaves were dipped in each solution for 10 s. The leaves were then air-dried and placed in Petri dishes (15 cm diameter). Deionized water served as the negative control. Each treatment included three biological replicates, with ten third-instar silkworms introduced per replicate. Mortality and sublethal effects were recorded at 24, 48, 72 and 96 h post-exposure (Sun et al., 2012).

#### 2.13.2 Zebrafish toxicity test

Acute toxicity to zebrafish was evaluated by exposing adult fish (*n* = 5 per replicate) to 7% ME at concentrations of 200, 100, 50, and 25 mg/L in 500 mL polypropylene containers (200 mL working volume, ddH_2_O). Mortality and behavioral abnormalities were monitored at 24, 48, and 72 h (Chen et al., 2025).

#### 2.13.3 Earthworm contact toxicity assay

Filter paper contact tests were conducted using 9 cm Petri dishes lined with filter paper impregnated with 7% ME solutions (200, 100, 50, and 25 μg/cm^2^). Three earthworms were introduced per dish, with three replicates per concentration. Mortality was assessed after 24, 48 and 72 h of exposure (Lukkari et al., 2005).

### 2.14 Data processing and analysis

The EC_50_ was determined through probit analysis of concentration-response curves. A linear regression model (*y* = a + b*x*) was applied, where *y* represents the probit-transformed inhibition percentage and *x* corresponds to the drug concentration. One-way ANOVA was first used to evaluate treatment effects. When significant differences were observed (*p* < 0.05), Tukey's HSD test was applied to compare group means. Disease Index (DI) and Relative Efficacy (RE) data were examined before analysis. Normality was tested using the Shapiro–Wilk test, and homogeneity of variance was assessed using Levene's test. RE is percentage data, and DI is ordinal data. These data types may not meet ANOVA assumptions. Therefore, arcsine square root transformation was applied before one-way ANOVA. Means were compared using Tukey's HSD test (*p* < 0.05). If the data still did not follow a normal distribution after transformation, a non-parametric test was used. The Kruskal–Wallis test was applied, followed by Dunn's post-hoc test. All statistical computations were performed using DPS software (v7.05).

## 3 Results and discussion

### 3.1 Optimization of extraction process for CLE

#### 3.1.1 Solvent and drying method selection

Preliminary screening revealed that air-dried leaves yielded extracts with superior inhibitory activity against *P. italicum* and *P. digitatum* compared to oven-dried leaves, though the latter exhibited marginally higher extraction yields. Extraction yield was calculated as: Yield (%) = (mass of dried extract / mass of dried leaf powder) × 100 (Table S1). Among solvents tested (anhydrous ethanol, petroleum ether, ethyl acetate), petroleum ether extracts showed the strongest antifungal activity against *P. italicum* but significantly lower yields (2.59–3.25%) than ethanol extracts (6.86–7.05%). Given the balance between efficacy and yield, anhydrous ethanol and air-dried leaves were selected for further optimization.

#### 3.1.2 Single-factor experiments

Single-factor experiments systematically evaluated key extraction parameters (Table S2). Anhydrous ethanol outperformed aqueous ethanol (75% and 50%) in both antifungal efficacy (50.00% inhibition of *P. italicum* and 100% for *P. digitatum* at 200 mg/L) and extraction yield (7.32%), justifying its selection as the optimal solvent (Table S3). Ultrasonic temperature significantly influenced yield, peaking at 60 °C (9.00%) before declining at higher temperatures, while antifungal activity remained consistent across temperatures (20–80 °C) (Table S4, Fig. S1A). Ultrasound time studies revealed maximal yield (11.3%) at 3 h without compromising inhibitory effects (Table S5, Fig. S1B). Similarly, a solvent-to-material ratio of 1:15 (m/v) was identified as optimal, balancing extraction efficiency (11.3% yield) and resource utilization (Table S6, Fig. S1C). These results collectively established baseline conditions for subsequent RSM optimization.

#### 3.1.3 Response surface methodology (RSM)

**Fig. 1:**
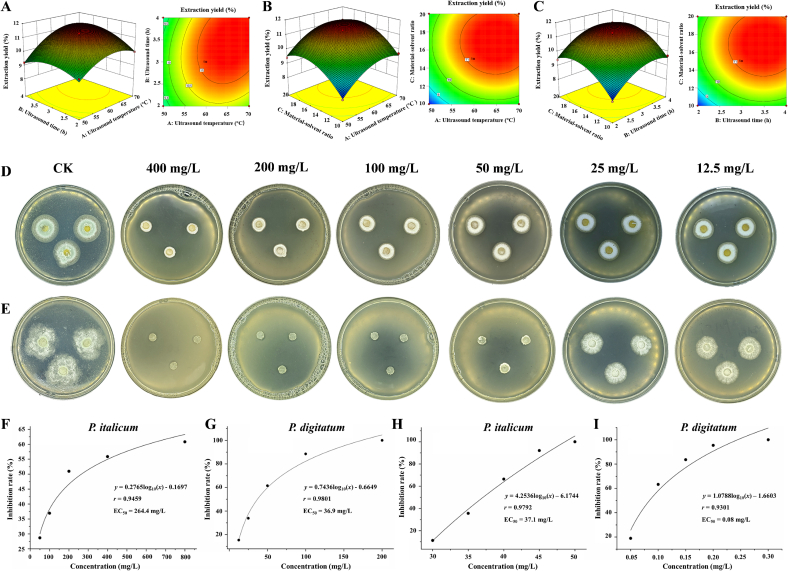
Combined graphical representation of process optimization and antifungal activity evaluation. (A-C) Response surface analyses of key extraction parameters influencing CLE yield; (D-E) Toxicity of CLE against *P. italicum* and *P. digitatum*; (F–I) The toxicity regression equation of CLE and TBZ against *P. italicum* and *P. digitatum*.

A Box-Behnken experimental design comprising 17 runs (12 factorial points and 5 center points, Table S7) was implemented to systematically optimize the extraction parameters. Through quadratic regression analysis using Design-Expert 10.0.4 (Table S8), we established a predictive mathematical model that describes the relationship between extraction parameters and yield. The second-order polynomial equation representing this relationship is expressed as:

$$Y\left(\%\right)=11.16+0.70A+0.28B+0.68C+0.30\mathrm{AB}+0.20\mathrm{BC}–0.73A^{2}–0.53B^{2}–0.88C^{2}$$

where Y represents the extraction yield (%), A is the ultrasonic temperature (°C), B is the extraction time (h), and C is the solvent-to-material ratio (m/v). All linear and quadratic terms of the three independent variables showed highly significant effects (*p* < 0.01), with the interaction term AB (temperature × time) also demonstrating significant influence (*p* < 0.05). The model's validity was confirmed by its high coefficient of determination (*r*^*2*^ = 0.93) and non-significant lack-of-fit (*F* = 0.90, *p* = 0.51). Response surface analysis identified optimal operational windows at 65–67 °C for temperature, 3.4–3.6 h for extraction time, and a solvent-to-material ratio of 1:16–1:18 (Fig. 1A-C). The close agreement between predicted (11.6%) and experimental (11.6%) yields at the rounded optimum conditions (66 °C, 3.4 h, 1:17 ratio) validated the model's reliability for industrial process optimization.

#### 3.1.4 Antifungal activity of optimized extracts

The optimized extract obtained under established conditions (66 °C, 3.4 h, 1:17 solvent ratio) demonstrated concentration-dependent antifungal activity against both *P. italicum* and *P. digitatum* (Fig. 1D-E). The EC_50_ values of the extract for *P. italicum* and *P. digitatum* were 264.4 mg/L and 37.1 mg/L, respectively. The EC_50_ values of TBZ for *P. italicum* and *P. digitatum* were 36.9 mg/L and 0.08 mg/L, respectively (Fig. 1F-I; Table S9). While the CLE demonstrates antifungal activity, its specific potency is significantly lower than that of the synthetic fungicide TBZ, requiring higher concentrations to achieve comparable inhibition. However, CLE offers important advantages as a sustainable biopesticide: being plant-derived, it provides a promising botanical alternative for postharvest disease management; its multiple bioactive constituents could reduce the risk of resistance development; and utilizing citrus leaf waste aligns with circular economy principles.

### 3.2 Chemical composition and active compound identification

GC–MS analysis of the optimized CLE identified 24 major constituents, including terpenoids, sterols, phenolics, and fatty acid derivatives, most compounds had spectral match factors above 90% (Table 1; Fig. S2), indicating a chemically diverse metabolite profile. Among these, five representative compounds—ethyl linoleate, phytol, 2,4-DTBP, *β*-caryophyllene, and squalene—were selected for *in vitro* antifungal screening based on a combination of criteria (Table 2): (1) representative coverage of distinct chemical classes (phenolics, sesquiterpenes, diterpenes, triterpenes, and fatty acid esters), (2) their notable relative peak areas in the GC–MS profile (Table 1), (3) previously reported membrane-active or antimicrobial potential, and (4) the commercial availability of high-purity authentic standards. Among five selected components screened for antifungal activity, 2,4-DTBP exhibited the strongest antifungal activity against both *P. italicum* (EC_50_ = 12.9 mg/L) and *P. digitatum* (EC_50_ = 6.5 mg/L), consistent with its structural features featuring hydrophobic alkyl substituents and a phenolic hydroxyl group that disrupt fungal membranes (Table S10). While 2,4-DTBP is widely recognized as a synthetic intermediate in the plastics industry, its occurrence as a natural secondary metabolite has been increasingly documented in various plant and microbial species, including *Pseudomonas sp.* and Pu-erh tea (Fan et al., 2023; Kushveer et al., 2023; Qi et al., 2021; Wang et al., 2025). Our rigorous use of pre-baked glass apparatus, absolutely negative solvent blanks, and consistent detection across multiple independent extraction batches confirm that the detected 2,4-DTBP is completely endogenous to the *Citrus reticulata* leaves. These controls strongly minimized the possibility of analytical contamination and support the endogenous occurrence of 2,4-DTBP in citrus leaves. Its high lipophilicity (LogP ∼5.1), conferred by the bulky *tert*-butyl groups, facilitates rapid partitioning into the fungal phospholipid bilayer, a trait shared by other membrane-disrupting phytochemicals. This aligns with recent studies identifying 2,4-DTBP as a potent antifungal agent in microbial metabolites (Fan et al., 2023), yet its specific role in citrus defense has been previously overlooked. However, it is crucial to acknowledge that in complex botanical extracts like CLE, the overall macroscopic antifungal efficacy is rarely attributable to a single molecule. The unselected or minor components likely exert synergistic or additive effects, potentially enhancing the membrane-disrupting capability or overall bioavailability of 2,4-DTBP. Therefore, 2,4-DTBP is proposed here as a principal active marker rather than the sole contributor to the efficacy of the 7% ME. This multicomponent synergy represents a fundamental advantage of botanical formulations, as it minimizes the risk of fungal resistance development compared to single-target synthetic fungicides. Although the present study focused on ‘Ponkan’ leaves from one representative production region, the standardized sampling strategy and pooled-leaf extraction were used to improve within-orchard consistency; future studies involving additional citrus cultivars and geographic origins would further verify the broader applicability of this approach.

**Table 1:** GC–MS Analysis Results of CLE.

Number	Retention time	Chemical constituents	Chemical Structure	CAS Number	Molecular Formula	Peak area (%)	Matching degree
1	12.839	*β*-elemene		515–13-9	C_15_H_24_	1.06	91
2	13.240	*β*-Caryophyllene		87–44-5	C_15_H_24_	0.29	99
3	13.692	*α*-Caryophyllene		6753-98-6	C_15_H_24_	0.24	97
4	14.111	*β*-selinene		17,066–67-0	C_15_H_24_	0.57	99
5	14.241	*α*-Selinene		473–13-2	C_15_H_24_	0.88	95
6	12.294	2,4-DTBP		96–76-4	C_14_H_22_O	1.02	95
7	15.225	Spathulenol		6750-60-3	C_15_H_24_O	0.88	99
8	15.303	9-epi-*β*-Caryophyllene		68,832–35-9	C_15_H_24_	1.38	91
9	15.609	2,4-Dimethyl-3-Cyclohexenecarboxaldehyde		68,039–49-6	C_9_H_14_O	0.77	92
10	15.799	Isospathulenol		88,395–46-4	C_15_H_24_O	1.04	99
11	15.925	Trimethylsilyl laurate		55,520–95-1	C_15_H_32_O_2_Si	0.50	98
12	16.166	(+)-Longifolene		475–20-7	C_15_H_24_	1.65	86
13	17.919	Neophytadiene		504–96-1	C_20_H_38_	1.50	99
14	18.522	Eicosane		112–95-8	C_20_H_42_	0.17	94
15	18.824	7,9-Di-*tert*-butyl-1-oxaspiro[4,5]deca-6,9-diene-2,8-dione		82,304–66-3	C_17_H_24_O_3_	0.69	94
16	19.142	Palmitic acid		57–10-3	C_16_H_32_O_2_	0.71	93
17	19.238	Dibutyl phthalate		84–74-2	C_16_H_22_O_4_	0.52	91
18	19.457	Ethyl palmitate		628–97-7	C_18_H_36_O_2_	2.13	98
19	20.667	Phytol		150–86-7	C_20_H_40_O	2.43	98
20	21.033	Ethyl linoleate		544–35-4	C_20_H_36_O_2_	0.81	99
21	21.100	Ethyl linolenate		1191-41-9	C_20_H_34_O_2_	1.32	99
22	21.296	Ethyl stearate		111–61-5	C_20_H_40_O_2_	0.60	97
23	23.302	2,2′-Methylenebis[4-methyl-6-*tert*-butylphenol]		119–47-1	C_23_H_32_O_2_	0.67	99
24	26.481	Squalene		111–02-4	C_30_H_50_	4.83	93

**Table 2:** Antifungal activity of partial constituents of CLE against *P. italicum* and *P. digitatum.*

Compound	Chemical Structure	Concentration (mg/L)	Inhibition rate (%)	
			*P. italicum*	*P. digitatum*
Ethyl linoleate		50	−14.3 ± 6.25 f	−0.02 ± 2.67 cd
		200	−12.3 ± 3.04 ef	−7.11 ± 4.15 d
Phytol		50	−10.2 ± 1.97 def	9.72 ± 1.40 b
		200	−8.13 ± 1.64 def	3.53 ± 4.01 bc
2,4-DTBP		50	83.7 ± 6.89 b	92.3 ± 0.81 a
		200	100.0 ± 0.00 a	100.0 ± 0.00 a
*β*-Caryophyllene		50	−10.2 ± 1.97 def	7.94 ± 4.52 bc
		200	0.00 ± 3.03 cd	0.00 ± 0.00 cd
Squalene		50	−2.08 ± 4.71 cde	0.05 ± 4.64 cd
		200	6.19 ± 5.47 c	8.82 ± 3.96 bc

Note: Data are presented as untransformed means ± SD. Statistical analyses were performed on arcsine square root-transformed inhibition-rate data where appropriate, followed by Tukey's HSD test (*p* < 0.05).

2,4-DTBP, a naturally occurring phenolic compound reported in various plant species, exhibits multiple biological activities, including antimicrobial, antioxidant, and anti-inflammatory effects, suggesting potential utility in both agrochemical and pharmaceutical applications (Guan et al., 2025; Ulloa et al., 2023). The presence of 2,4-DTBP in CLE was confirmed by HPLC–MS using an authentic standard, as evidenced by an identical retention time and a matching molecular ion at *m/z* 205.2 ([M–H]^−^), thereby confirming its presence in the citrus leaf extract (Fig. S3A—D). The blank solvent control showed no detectable signal, indicating negligible contamination during extraction. Previous reports have identified 2,4-DTBP in several higher plants, including *Eucalyptus* and *Cinnamomum* species, supporting its classification as a naturally occurring phytochemical (Fan et al., 2023).

### 3.3 Physical properties and stability of 7% ME and evaluation of antifungal activity against Penicillium species

#### 3.3.1 Development and optimization of 7% ME

The microemulsion formulation of CLE was systematically optimized to enhance its antifungal efficacy against *P. italicum* and *P. digitatum*. Solvent screening revealed that anhydrous ethanol exhibited superior solubility for the extract, with no precipitation observed after 72 h at 4 °C, making it the optimal organic solvent for microemulsion preparation (Table S11). The hydrophilic-lipophilic balance (HLB) value was critical for stability, with HLB 12–16 identified as the optimal range for oil-in-water (O/W) microemulsions (Fig. S5; Table S12-S13). Among the tested binary surfactant systems, the combination of CDBS and Tween-20 showed the best overall performance in terms of formulation clarity, dilution stability, and antifungal activity. Therefore, CDBS and Tween-20 were selected as the optimized surfactant pair for preparing the final 7% ME, with a mass ratio of 2:8, corresponding to 4% CDBS and 16% Tween-20 in the final formulation (Table S14-S15). Preliminary concentration gradient screenings indicated that 7% (*w*/*w*) was the maximum solubilization capacity for CLE in this specific system; exceeding this concentration resulted in turbidity and phase separation. The final microemulsion formulation (7% ME) was a transparent, homogeneous, dark brown liquid with no turbidity or crystallization at room temperature, composed of 7% CLE, 27% anhydrous ethanol, 4% CDBS, 16% Tween-20, and 46% water. It had a pH of 6.6 ± 0.20 and a psize range of 37.8–67.6 nm (Fig. S4, Table 3).

**Table 3:** Quality control specifications for 7% ME.

Technical indicator	Result
Surface	Transparent uniform
Size (nm)	37.8–67.6
Transparent temperature range (°C)	−20–80
pH	6.6
Viscosity	11 mPa·s
Spray ability	Qualification
Light stability	Qualification
Dilution stability	Qualification
Emulsion stabilization	Qualification
Low-temperature stability (0°C)	Qualification
Thermal storage stability (54 ± 2°C)	Qualification
Persistent foaming capacity (mL)	18
Type of 7% Microemulsion	O/W

#### 3.3.2 Physical properties, stability and antifungal activity of 7% ME

Quality assessments confirmed the 7% ME's stability under diverse conditions (Table 3). It remained transparent across a broad temperature range (−20 °C to 80 °C) and exhibited no phase separation after dilution with standard hard water. Cold storage (0 °C for 7 d) and accelerated thermal stability tests (54 °C for 14 d) revealed no significant changes in physical properties or antifungal activity (Table S16). The 7% ME also met industrial standards for foam persistence (18 mL) and centrifugal stability (0.15 mL precipitate). Additional application-oriented characterization further supported the suitability of 7% ME as a sprayable stock formulation (Table 3). The undiluted 7% ME exhibited an apparent viscosity of 11 mPa·s at 25 ± 1 °C, indicating low viscosity and good fluidity for spray application. After dilution with standard hard water at ratios of 1:100, 1:200, and 1:500, all working solutions remained clear and transparent after storage at 25 °C for 24 h. No visible particles, precipitation, or phase separation were observed, even after shaking. In the sprayability test, all diluted working solutions were continuously sprayed 50 times using a handheld sprayer, and no nozzle clogging, visible particulate matter, or interruption of spray flow was observed. Furthermore, after exposure to continuous illumination at 4000–6000 lx for 24 h, the 7% ME still remained clear and transparent, with no visible particles, precipitation, or phase separation. Similar appearance was observed in the aluminum-foil-wrapped dark control. These results indicate that 7% ME can be prepared as a concentrated stock formulation and diluted before use, while maintaining short-term dilution stability, light stability, and spray compatibility.

These results underscore the potential of 7% ME as a sustainable alternative to synthetic fungicides for postharvest citrus preservation (Table 3). The optimized 7% ME formulations were tested for antifungal activity against *P. italicum* and *P. digitatum* after meeting the required quality criteria. It is noteworthy that microemulsion significantly enhanced the antifungal efficacy of CLE. The EC_50_ values against *P. italicum* and *P. digitatum* were reduced to 69.3 mg/L and 25.2 mg/L, respectively — nearly a fourfold improvement compared to the original CLE (Table S17). In contrast, the blank ME adjuvant control matrix—strictly formulated to match the 7% ME by replacing the 7% CLE with an equivalent mass of water (comprising 27% anhydrous ethanol, 4% CDBS, 16% Tween-20, and 53% water)—exhibited negligible inhibitory activity against both fungal species (Table S17). This perfectly matched control confirms that the observed antifungal efficacy is exclusively driven by the botanical active ingredients. These results demonstrate that the optimized 7% ME is a stable, effective, and environmentally compatible biofungicide with strong potential for practical application in postharvest citrus protection.

### 3.4 The biocontrol effectiveness of 7% ME against postharvest disease on citrus fruit

**Fig. 2:**
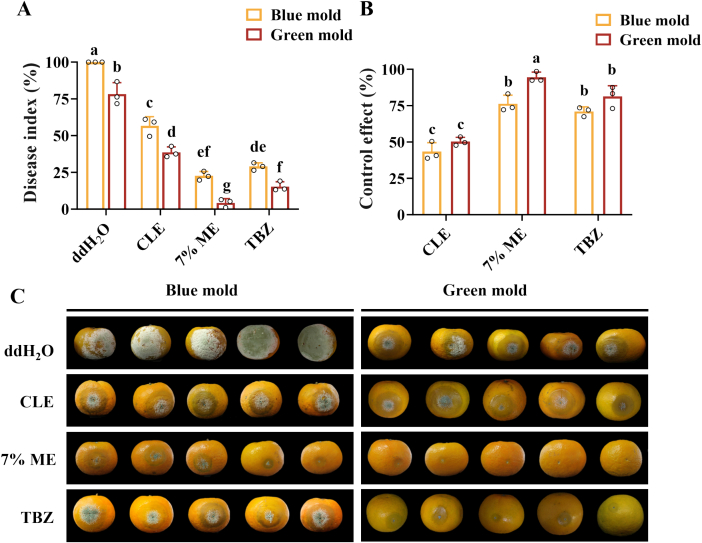
*In vivo* control efficiency of 7% ME against blue mold and green mold. (A) Statistical results of disease index for blue mold and green mold; (B) Statistical analysis of *in vivo* disease control efficacy of 7% ME against blue mold and green mold; (C) Visual representation of the *in vivo* disease control efficacy of 7% ME against blue mold and green mold on citrus fruits. Data are presented as untransformed means ± SD of three biological replicates. Statistical analyses were performed on arcsine square root-transformed data where appropriate. Different lowercase letters indicate significant differences among treatments within the same pathogen according to Tukey's HSD test (*p* < 0.05). (For interpretation of the references to colour in this figure legend, the reader is referred to the web version of this article.)

The *in vivo* efficacy trials revealed the superior performance of a novel 7% ME in controlling postharvest citrus molds. The CLE showed 43.7% control efficacy against blue mold caused by *P. italicum* and 50.6% against green mold caused by *P. digitatum*. Remarkably, the 7% ME formulation achieved significantly higher disease suppression, with 77.4% control of blue mold and an exceptional 94.5% control of green mold. When evaluated at biologically equipotent doses (5 × EC_50_), this performance rivaled and even exceeded the practical control levels of the conventional fungicide TBZ, which showed 71.0% and 81.3% control efficacy against blue and green mold, respectively (Fig. 2A-C; Table S18). While the absolute application concentration of 7% ME was higher than that of the pure synthetic TBZ, these results indicate that the microemulsion delivery system provides a remarkably high ceiling for *in vivo* disease suppression. The 7% ME's outstanding efficacy, particularly its near-complete inhibition of green mold, can be attributed to its optimized delivery system that enhances the solubility, stability and bioavailability of bioactive compounds. With its demonstrated dual-pathogen efficacy, plant-based origin and reduced environmental impact, this 7% ME represents a high-performance, sustainable alternative to synthetic fungicides for citrus postharvest protection, highlighting how advanced formulation technologies can maximize the potential of botanical antimicrobials.

### 3.5 Quantification of defense enzyme activities in citrus

**Fig. 3:**
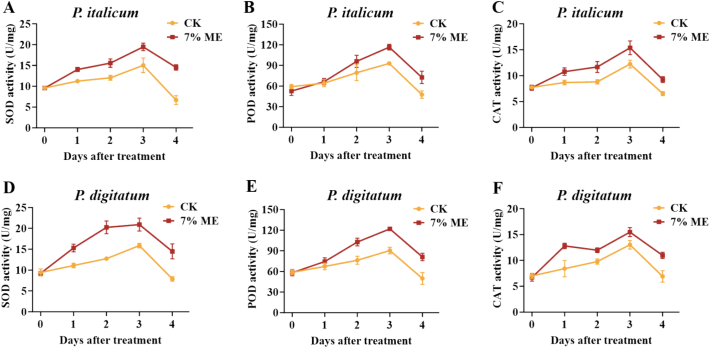
Alterations in defense-related enzyme activities in citrus fruit treated with 7% ME. (A-C) SOD, POD, and CAT activities in citrus peel inoculated with *P. italicum* at 0–4 days post-treatment. (D–F) Enzyme activities in peel inoculated with *P. digitatum.* Values represent the mean ± SD of three independent biological replicates.

Quantitative analysis of defense enzyme activities in citrus fruit following 7% ME treatment was conducted *in vivo* (Fig. 3). Compared to the control, application of 7% ME increased the activities of SOD, CAT, and POD, indicating the induction of systemic defense responses against the infection caused by *P. italicum* (blue mold) and *P. digitatum* (green mold). Enzyme activities increased progressively from the onset of treatment. No significant differences were observed among groups at 0 h, confirming uniform baseline conditions. Peak activities of SOD, CAT, and POD were reached at 72 h post-inoculation, followed by a slight decline at 96 h. Notably, in 7% ME-treated fruit, CAT and POD activities were significantly higher in those challenged with *P. digitatum* than with *P. italicum*. This suggests that 7% ME more effectively elicited defense-related enzyme responses against green mold infection. The sustained elevation of SOD and CAT suggests that the microemulsion components, potentially including bioavailable 2,4-DTBP, may act as abiotic elicitors that enhance antioxidant defense responses in citrus fruit. The sustained elevation of these enzymes correlates with an enhanced physiological resistance against hyphal penetration. However, since the current study relies on phenotypic enzyme assays, further validation employing specific inhibitors or gene silencing is required to determine whether this enhancement is a direct elicitation by the microemulsion or an indirect stress response (Cheng et al., 2020).

### 3.6 Microscopic evidence of 7% ME-induced structural damage in *P. italicum* and *P. digitatum* hyphae

**Fig. 4:**
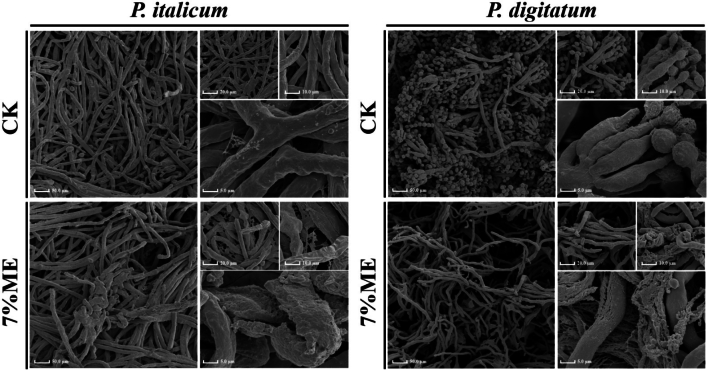
SEM of *P. italicum* and *P. digitatum* hyphae in the untreated control and 7% ME-treated group. The multiple panels for each treatment represent randomly selected, distinct regions captured at varying magnifications to demonstrate the widespread and representative nature of the morphological alterations.

SEM analysis revealed significant alterations in the hyphal morphology of *P. italicum* and *P. digitatum* following treatment with 7% ME. Due to inherent differences in their sporulation kinetics at the 72 h sampling point, *P. italicum* predominantly presented vegetative hyphal networks, whereas *P. digitatum* exhibited abundant reproductive conidiophores at the colony margins. In the control groups, these respective structures exhibited a smooth, intact, and uniform surface with regular architecture and continuous growth fronts (Fig. 4). In contrast, both the vegetative hyphae of *P. italicum* and the conidiophores of *P. digitatum* treated with 7% ME displayed severe morphological distortions. Notably, these structures appeared shrunken or ruptured, with visible cracks and pore formation on the surface, particularly at the apical regions (Fig. 4). These widespread morphological aberrations suggest that the membrane-disrupting action of 7% ME is highly robust, indiscriminately compromising the integrity of the fungal cell envelope (across different developmental structures), resulting in increased permeability and leakage of intracellular constituents. Although SEM primarily provides surface-level information, the extensive deformation and rupture of hyphae observed here are consistent with cell-envelope disruption mechanisms reported for other plant-derived antifungal agents.

### 3.7 Effect of 7% ME on cell membrane permeability

**Fig. 5:**
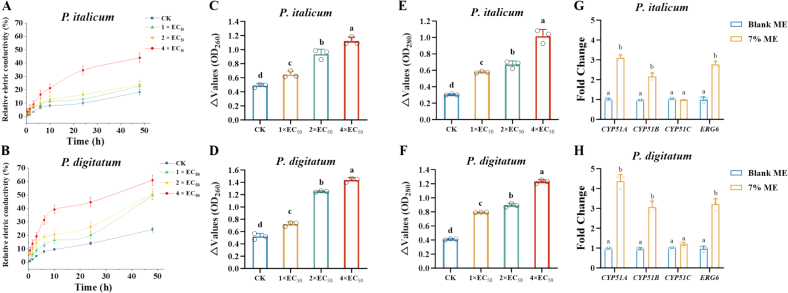
Increased membrane permeability, leakage of intracellular components, and transcriptional responses in *P. italicum* and *P. digitatum* induced by 7% ME treatment. (A–B) Changes in cell membrane permeability of *P. italicum* and *P. digitatum* after treatment with 7% ME; (C–F) Release of nucleic acids and proteins from *P. italicum* and *P. digitatum* after treatment with 7% ME; (G-H) Relative expression levels (fold change) of ergosterol biosynthesis-related genes (*CYP51A*, *CYP51B*, *CYP51C*, and *ERG6*) in *P. italicum* and *P. digitatum* following a 6 h exposure to the 7% ME or Blank ME control. In panels A–F, different curves or bars represent increasing concentrations of 7% ME. In panels G and H, bars represent Blank ME and 7% ME treatments. Error bars represent SD of three biological replicates. Different lowercase letters indicate statistically significant differences among treatments within each panel (*p* < 0.05).

The antifungal mechanism of 7% ME was investigated through membrane integrity assessment, given the critical role of cellular membranes in maintaining fungal viability and structural integrity. The ergosterol-enriched plasma membrane serves as a critical barrier; disruption causes rapid cell lysis and irreversible physiological damage in fungi. In this context, changes in membrane permeability serve as a reliable indicator of membrane-targeting activity. Relative conductivity measurements revealed a distinct dose- and time-dependent disruption of membrane integrity in both *P. italicum* and *P. digitatum* following treatment with 7% ME. During the initial 12–24 h of exposure, a gradual increase in electrolyte leakage was observed, indicative of progressive perturbation of the lipid bilayer and the formation of transient pores or defects. Notably, a sharp escalation in relative conductivity occurred after 24 h, suggesting a threshold effect where accumulated damage culminates in catastrophic membrane failure and massive efflux of intracellular contents. This irreversible damage phase distinguishes 7% ME from weaker fungistatic agents. The pattern of delayed but rapid electrolyte leakage mirrors the mode of action of lipophilic terpenes like carvacrol, which accumulate in the lipid bilayer until membrane fluidity is critically compromised (Zhang et al., 2019). This phase transition was particularly pronounced in *P. digitatum*, which exhibited greater sensitivity compared to *P. italicum* (Fig. 5A-B).

A clear concentration-dependent response was observed, whereby higher concentrations of 7% ME resulted in more rapid and pronounced membrane damage, as indicated by the accelerated electrolyte leakage. This trend strongly suggests that the antifungal activity of 7% ME is mediated through direct disruption of membrane integrity, ultimately leading to the loss of cellular homeostasis. This proposed mechanism is consistent with previous reports on lipophilic antifungal agents that compromise membrane barrier function (Zhang et al., 2019). The differential sensitivity between species (*P. digitatum* > *P. italicum*) may reflect variations in membrane composition or repair capacity, warranting further investigation. These findings establish membrane disruption as a primary mode of action underlying the efficacy of 7% ME against postharvest citrus pathogens, thereby supporting its potential as a biofungicide with a well-defined mechanism of action.

### 3.8 Effect of 7% ME on release of cellular contents

The integrity of the fungal cell membrane was assessed by measuring the leakage of intracellular components, including nucleic acids and proteins. The concentrations of nucleic acids and proteins released from *P. italicum* and *P. digitatum* mycelial suspensions were quantified by measuring the absorbance at 260 nm and 280 nm, respectively. As shown in Fig. 5C-F, following treatment with various concentrations of 7% ME for 6 h, nucleic acids and proteins were released from the mycelial cells of *P. italicum* and *P. digitatum* to varying extents, in a concentration-dependent manner. In addition, all samples treated with 7% ME exhibited significantly higher absorbance values at both 260 nm and 280 nm compared to the corresponding control groups. Notably, the extent of membrane leakage in *P. digitatum* mycelium was greater than that observed in *P. italicum* mycelium. Thus, these results indicated that the cell membrane integrity of *P. italicum* and *P. digitatum* was damaged by 7% ME.

### 3.9 Transcriptional validation of ergosterol pathway targeting

While the physiological assays (Fig. 5A-F) indicated that 7% ME compromises fungal cell envelopes, we further examined whether genes associated with ergosterol biosynthesis were transcriptionally responsive to 7% ME treatment. Consequently, the relative expression levels of the sterol 14α-demethylase paralogous genes (*CYP51A*, *CYP51B*, and *CYP51C*) and the downstream sterol 24-*C*-methyltransferase (*ERG6*) were quantified using qRT-PCR following a 6 h exposure to the 7% ME.

As illustrated in Fig. 5G and H, the Blank ME vehicle control effectively normalized the background noise originating from the solvent and surfactants, maintaining a baseline expression level of approximately 1 across all tested genes. In contrast, treatment with 7% ME induced marked transcriptional changes in several ergosterol biosynthesis-related genes in both *Penicillium* species. Specifically, *CYP51A* exhibited the most dramatic upregulation, increasing by roughly 3.1-fold in *P. italicum* and 4.4-fold in *P. digitatum*. Concurrently, the *CYP51B* paralog and the downstream *ERG6* were also significantly upregulated (approximately 2- to 3-fold) in both pathogens. Interestingly, the expression of *CYP51C* remained largely unaltered, underscoring the specificity of the cellular response.

This robust transcriptional burst represents a classic compensatory feedback mechanism characteristic of filamentous fungi under chemical stress. When exogenous agents (such as 2,4-DTBP in the formulation) interact with the enzymatic function of existing CYP51 proteins, the fungal cells inevitably face disturbance of sterol homeostasis. To overcome this blockade and survive the ensuing membrane instability, the sterol regulatory networks are forcefully activated, driving the extensive overexpression of primary target genes (*CYP51A* and *CYP51B*) to synthesize more enzyme, while simultaneously upregulating downstream genes (*ERG6*) to salvage the sterol pathway. These mycelial transcriptional data are consistent with the physiological membrane-damage assays and suggest that 7% ME may disturb fungal membrane homeostasis, accompanied by a compensatory transcriptional response involving ergosterol biosynthesis-related genes.

### 3.10 Molecular docking studies

**Fig. 6:**
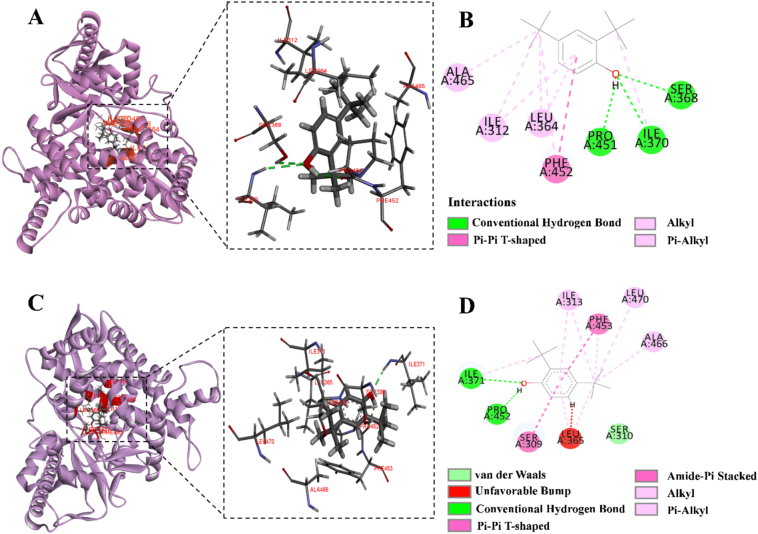
Molecular docking analysis of 2,4-DTBP with CYP51 proteins of *P. italicum* (Q12664) and *P. digitatum* (A1XG20). (A) The 3D binding conformation of 2,4-DTBP in the active pocket of *P. italicum* CYP51 (UniProt: Q12664); (B) The 2D interaction diagram showing key amino acid residues and bonding types (hydrogen bonds, alkyl, and Pi-alkyl interactions) in *P. italicum*; (C) The 3D binding conformation of 2,4-DTBP in the active pocket of *P. digitatum* CYP51 (UniProt: A1XG20); (D) The 2D interaction diagram showing key amino acid residues in *P. digitatum*.

The membrane leakage assays, together with the transcriptional responses of *CYP51* and *ERG6* genes, suggested that ergosterol biosynthesis-related membrane homeostasis may be involved in the antifungal action of 7% ME. To explore a possible molecular interaction between the active marker 2,4-DTBP and CYP51A, molecular docking was employed to simulate its interaction with the CYP51A isoforms (UniProt: Q12664 for *P. italicum* and A1XG20 for *P. digitatum*) (Oreiro et al., 2025). This target selection is critical for food safety applications: unlike mammalian cells, which rely on cholesterol for membrane structure, fungal membranes are strictly dependent on ergosterol. Therefore, targeting CYP51 exploits this fundamental biological divergence, offering a strategy for selective toxicity against *Penicillium* pathogens with minimal risk to human health. Inhibition of CYP51 disrupts membrane fluidity and integrity, leading to growth suppression in *Penicillium* species (Niu et al., 2025; Zhang et al., 2023b). Docking simulations revealed that 2,4-DTBP binds stably to the active sites of *P. italicum* (Q12664) and *P. digitatum* (A1XG20) CYP51A, with calculated binding affinities of −7.39 kcal/mol and − 5.47 kcal/mol, respectively (Fig. 6 A-D; Table S19). In *P. italicum*, 2,4-DTBP formed hydrogen bonds with Pro451, Ile370 and Ser368, together with π–π stacking against Phe452 and hydrophobic interactions involving Leu364, Ile312, and Ala465. In *P. digitatum*, hydrogen bonds with Ile371 and Pro452, involving Phe453 and Ser309, and hydrophobic contacts with Leu365, Leu470, Ile313, and Ala466 contributed to ligand stabilization. Molecular docking simulations provided atomistic insight into this membrane disruption. The docking pose suggested that the bulky tert-butyl groups of 2,4-DTBP may fit into a hydrophobic pocket near the substrate access region of CYP51A, where they form hydrophobic contacts with residues such as Leu364 and Ala465. This interaction pattern raises the possibility that 2,4-DTBP may interfere with substrate accommodation or access to the catalytic region. However, direct biochemical assays are still required to confirm whether 2,4-DTBP inhibits CYP51 enzymatic activity and affects ergosterol production in fungal cells (Zhang et al., 2023b). This computational model predicts that 2,4-DTBP could theoretically act as a competitive inhibitor blocking the substrate access channel, providing a plausible molecular mechanism for the observed fungicidal effects.

These results indicate that 2,4-DTBP exhibits stable binding to CYP51A primarily mediated by hydrophobic and aromatic contacts, supported by hydrogen bonds anchoring the ligand within the catalytic pocket. The presence of similar interacting residues in both fungi suggests a potentially conserved binding pattern, which may partly explain the broad-spectrum antifungal activity observed in biological assays. Together, the predicted binding affinities and residue-level interactions provide a computational hypothesis that 2,4-DTBP may interact with CYP51A. While physiological assays confirmed actual membrane disruption, our transcriptional data provide the crucial molecular link to the ergosterol pathway. Supported by the physiological and transcriptional findings, the docking results suggest CYP51A as a plausible candidate target associated with the antifungal activity of 2,4-DTBP, although *in vitro* enzymatic assays and ergosterol quantification are needed for direct validation.

### 3.11 Safety assessment of 7% ME in non-target organisms

**Fig. 7:**
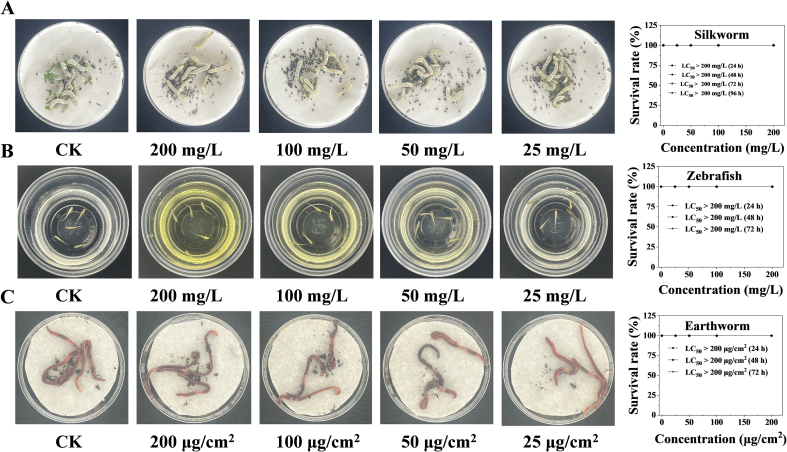
Safety evaluation of 7% ME in non-target organisms. (A) Silkworm, (B) Zebrafish, (C) Earthworm.

To evaluate the ecotoxicological safety of the citrus-derived 7% ME, acute toxicity assays were conducted on three representative non-target organisms: the silkworm (*B. mori*), zebrafish (*D. rerio*), and earthworm (*E. fetida*), representing terrestrial, aquatic, and soil-dwelling species, respectively. As shown in Fig. 7, no mortality or observable sublethal effects (*e.g.*, behavioral abnormalities, reduced mobility, or morphological changes) were recorded in any of the test organisms across all concentrations during the 72 or 96 h observation period. Survival rates remained consistently at 100% in both treatment and control groups. Although the formulation contains synthetic surfactants (*e.g.*, Tween-20) and ethanol, these adjuvants are widely accepted in agricultural applications. Tween-20 is considered a low-toxicity, biodegradable additive, and the ethanol content rapidly evaporates or degrades, posing minimal long-term environmental risk compared to persistent chemical fungicide residues.

These results indicate that 7% ME caused no acute toxicity to the tested non-target organisms under the current laboratory conditions, supporting its preliminary safety as a botanical postharvest treatment. It should be noted, however, that 2,4-DTBP residues in treated fruit and its long-term accumulation behavior in plant or soil systems were not evaluated in the present study and should be further examined before large-scale application.

## 4 Conclusions

This study proposes a circular-economy approach for citrus postharvest protection by transforming underutilized citrus leaf biomass into a bio-based fungicide. The optimized CLE, containing 2,4-DTBP as a principal active marker, was successfully formulated into a stable 7% ME with improved solubility and antifungal efficacy. The formulation demonstrated strong *in vivo* efficacy, particularly against *P. digitatum*, with a control efficacy of 94.5%, and provided disease suppression comparable to thiabendazole under the tested 5 × EC_50_ application conditions. Mechanistic investigations suggested a dual mode of action involving disruption of fungal membrane integrity, with qRT-PCR and computational docking supporting CYP51-associated ergosterol homeostasis as a plausible mechanistic link, together with enhanced phenotypic activities of host defense-related enzymes. Furthermore, the formulation showed no acute toxicity to the tested non-target organisms, including silkworms, zebrafish, and earthworms, under the current laboratory conditions. Collectively, these findings provide a scientifically supported framework for upcycling agricultural biomass into effective and sustainable postharvest protection agents.

## CRediT authorship contribution statement

**Lan-Tu Xiong:** Writing – review & editing, Writing – original draft, Visualization, Validation, Supervision, Software, Resources, Methodology, Investigation, Formal analysis, Data curation, Conceptualization. **Jing Yi:** Writing – original draft, Visualization, Validation, Supervision, Software, Resources, Project administration, Methodology, Investigation, Formal analysis, Data curation. **Jiayu Xu:** Validation, Investigation, Formal analysis. **Gen Zhang:** Visualization, Validation, Supervision, Software, Resources, Methodology, Conceptualization. **Yi-Lin Ma:** Methodology, Conceptualization. **Jing Jiao:** Writing – review & editing, Writing – original draft, Funding acquisition. **Ri-Yuan Tang:** Writing – review & editing, Writing – original draft, Visualization, Validation, Funding acquisition, Formal analysis, Data curation, Conceptualization.

## Declaration of competing interest

The authors declare that they have no known competing financial interests or personal relationships that could have appeared to influence the work reported in this paper.

## Data Availability

Data will be made available on request.
