# Supplementary material for: Citrus-leaf microemulsion controls postharvest mold through membrane disruption and host defense enhancement
Source: Food Chem X. 2026 Jun 5;37:104048. doi: 10.1016/j.fochx.2026.104048 (PMC13265713; doi:10.1016/j.fochx.2026.104048)
Supplement: Supplementary file 1 — Supplementary material [file mmc1.pdf]

Supporting information

Citrus-Leaf Microemulsion Controls Postharvest Mold through Membrane Disruption and Host Defense Enhancement

Lan-Tu Xiong[a,b,1], Jing Yi[a,1], Jiayu Xu[c], Gen Zhang[a], Yi-Lin Ma[a], Jing Jiao[d,]*, Ri-Yuan Tang[a,b,]*

[a] Key Laboratory of Advanced Materials for Facility Agriculture, Ministry of Agriculture, College of Materials and Chemical Engineering, South China Agricultural University, Guangzhou, 510642, P. R. China.

[b] State Key Laboratory of Green Pesticide, Integrative Microbiology Research Centre, Guangdong Provincial Key Laboratory of Microbial Signals and Disease Control, College of Plant Protection, South China Agricultural University, Guangzhou 510642, China.

[c] The Second Clinical Medical College, Southern Medical University, Guangzhou 510515, China.

[d] School of Chemistry and Civil Engineering, Shaoguan University, Shaoguan 512005, China

[1] These authors contributed equally to this work.

* Corresponding author.

E-mail address: jiaojing@sgu.edu.cn; rytang@scau.edu.cn.

Table of Contents

1.1 Plant material, reagents, and solvents 1

Figure S1. Evaluation of ultrasonic temperature (a), ultrasonic time (b), and solvent ratio (c) on extraction yield and antifungal activity. 2

Figure S2. Total ion chromatogram (TIC) of CLE analyzed by GC–MS. 2

Figure S3. HPLC and LC-MS Analysis of CLE and 2,4-DTBP Standard. Panels A and C show the HPLC chromatogram and LC-MS spectrum of CLE, respectively; Panels B and D present the corresponding data for the 2,4-DTBP standard.. 3

Figure S4. Particle size distribution of 7% ME was measured by DLS at 25℃ 3

Figure S5. Visual assessment of CLE clarification in surfactants across a range of HLB values. Panels A–I illustrate the CLE's appearance prior to dilution (HLB 8 ~ 16), while Panels J–O present the results after water dilution (HLB 11 ~ 16). 4

Table S1. Impact of extraction parameters on antifungal activity and yield of CLE.. 5

Table S2. Response surface methodology experimental design for factors 5

Table S3. Ethanol concentration modulates antifungal activity and extract yield of CLE 6

Table S4. Ultrasound temperature modulates antifungal activity and extract yield of CLE 6

Table S5. Antifungal activity and extract yield of CLE as a function of ultrasound treatment time.. 7

Table S6. Antifungal activity and extract yield of CLE as a function of solvent-to-material ratio.. 7

Table S7. Results of Box-Behnken experimental design.. 8

Table S8. Table of significance test for regression coefficients 9

Table S9. Toxicity of the different compounds against *P. italicum* and *P. digitatum*............ 10

Table S10. Toxicity of the 2,4-DTBP against *P. italicum* and *P. digitatum*.. 10

Table S11. Solvent screening results 11

Table S12. The clarifying effect of CLE in emulsifiers with different HLB values............... 11

Table S13. Binary surfactant combinations with HLB values greater than 12....................... 11

Table S14. Screening results of binary surfactants.. 12

Table S15. Inhibitory rate of 30% binary surfactant-based microemulsions against *P. italicum* and *P. digitatum* at 200 mg/L.. 12

Table S16. Inhibitory activity of 7% ME against *P. italicum* and *P. digitatum* at 200 mg/L before and after heat storage 12
Table S17. Toxicity of 7% ME against *P. italicum* and *P. digitatum* 13
Table S18. *In vivo* control efficiency of 7% ME against green mold and blue mold 13
Table S19. Molecular docking analysis of 2,4-DTBP with CYP51 proteins of *P. italicum* (Q12664) and *P. digitatum* (A1XG20) 13
Table S20. Primers used for qRT-PCR analysis in *P. italicum* 14
Table S21. Primers used for qRT-PCR analysis in *P. digitatum* 14

1.1 Plant material, reagents, and solvents

The sampled orchard was a conventional commercial "Ponkan" orchard consisting of mature fruit-bearing trees approximately 8–10 years old. Trees were planted at a spacing of approximately 3 m × 4 m, corresponding to a planting density of about 830 trees ha^{-1}. Lanshan County has a humid subtropical monsoon climate, with an average annual rainfall of approximately 1500–1600 mm. The orchard was managed under local conventional horticultural practices, including annual basal application of organic fertilizer after fruit harvest and supplementary compound fertilizer during the spring shoot and fruit enlargement stages. To reduce variation caused by leaf developmental stage, canopy height, and light exposure, the sampling criteria were standardized as far as possible: healthy, disease-free mature leaves of similar size (approximately 6–8 cm in length and 3–4 cm in width) were selectively harvested from the middle canopy at approximately 1.5 m above ground level. Leaves were collected from different canopy orientations and pooled before extraction to reduce within-orchard heterogeneity. Leaves with visible disease symptoms, insect damage, senescence, or mechanical injury were excluded. Potato dextrose agar (PDA) medium was purchased from Haibo Biotechnology Co. Analytical standards including Ethyl Linoleate, *β*-Sitosterol, and 2,4-di-tert-butylphenol (2,4-DTBP, >99% purity) were purchased from Bide Pharmatech Ltd. (Shanghai, China). To preclude phthalate or plasticizer contamination during extraction and analysis, all glassware was pre-washed with acetone and baked at 180°C for 4 h. The use of plastic materials (e.g., pipette tips, centrifuge tubes) was strictly avoided during solvent extraction steps involving organic solvents. Acetone, Methanol (chromatography grade), Dimethyl Sulfoxide, Anhydrous Ethanol (analytical grade), acquired from Shanghai Titan Technology Co., Ltd.; the surfactants used for microemulsion screening and preparation, including calcium dodecylbenzenesulfonate (CDBS, CAS: 26264-06-2, HLB = 4.5, purity >95%), Tween-20 (T-20, CAS: 9005-64-5, HLB = 16.7, purity >99%), Tween-80 (T-80), 1601#, and 1602#, were purchased from Hai'an Petroleum Chemical Factory (Jiangsu, China); Gas Chromatography-Mass Spectrometry Quadrupole System, produced by Agilent Technologies, Inc.; LC-100 Liquid Chromatography System, provided by Shanghai Wufeng Scientific Instrument Co., Ltd.; AB Sciex API3200 Liquid Chromatography-Mass

Spectrometry System, manufactured by AB Applied Biosystems, Inc.

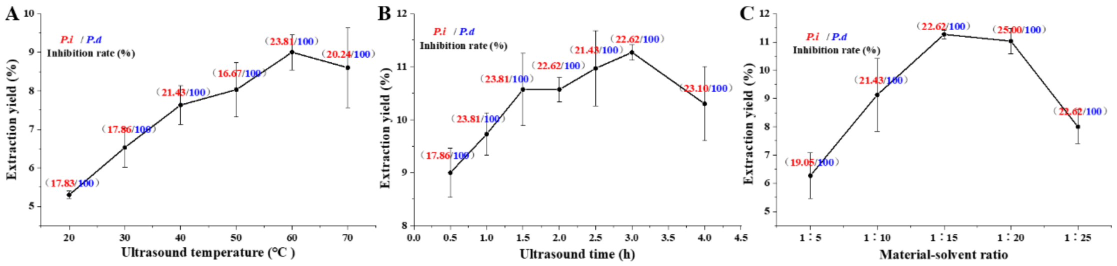

Figure S1. Evaluation of ultrasonic temperature (a), ultrasonic time (b), and solvent ratio (c) on extraction yield and antifungal activity.

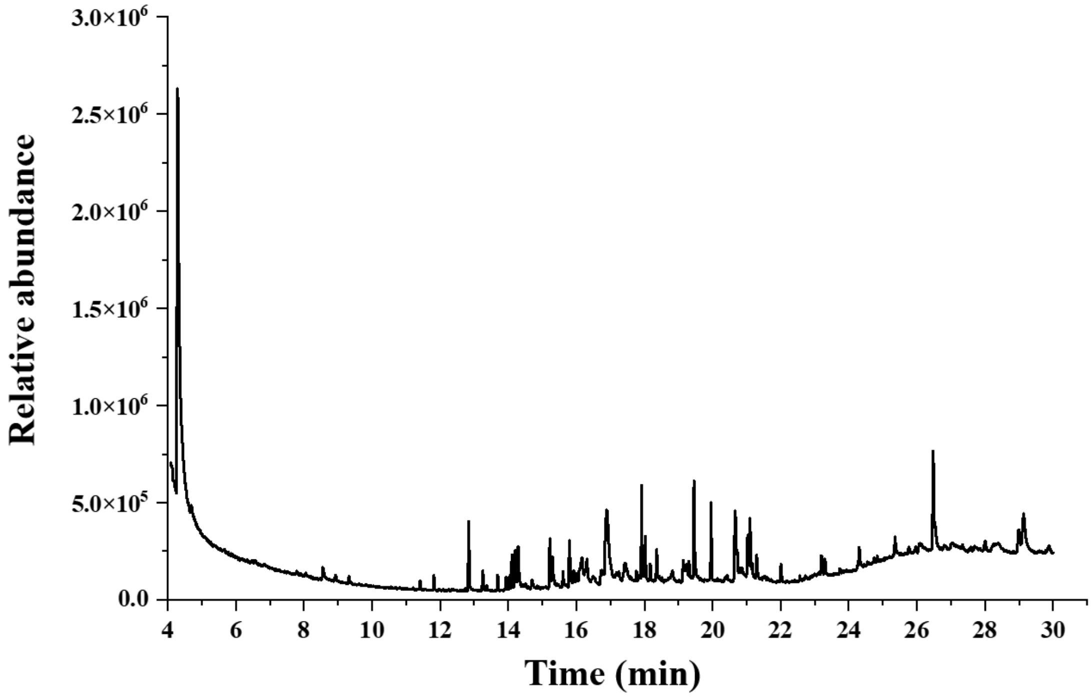

Figure S2. Total ion chromatogram (TIC) of CLE analyzed by GC–MS.

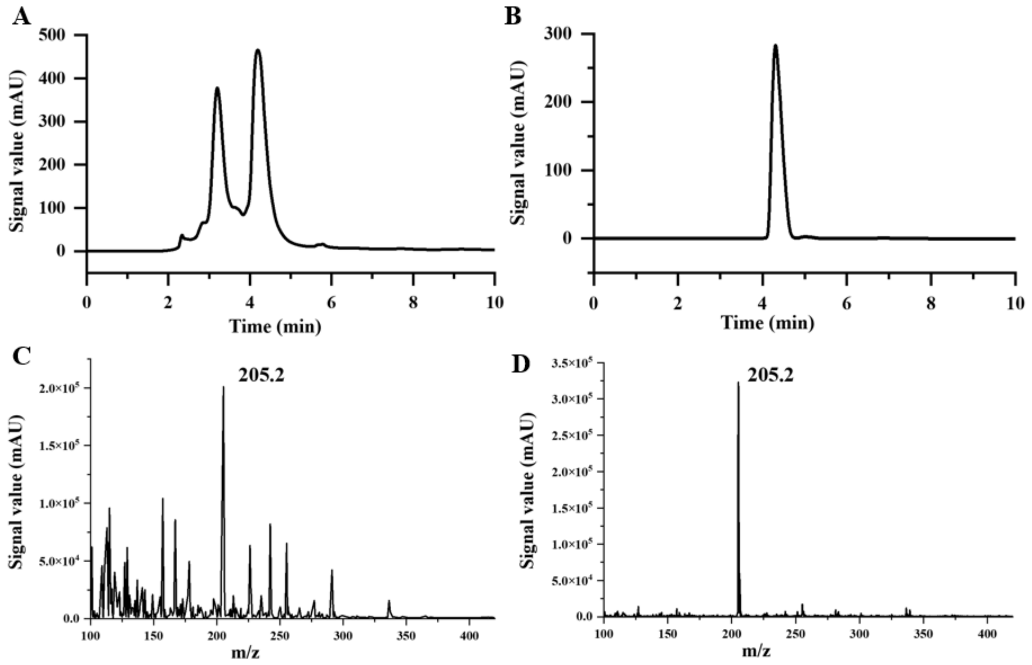

Figure S3. HPLC and LC-MS Analysis of CLE and 2,4-DTBP Standard. Panels A and C show the HPLC chromatogram and LC-MS spectrum of CLE, respectively; Panels B and D present the corresponding data for the 2,4-DTBP standard.

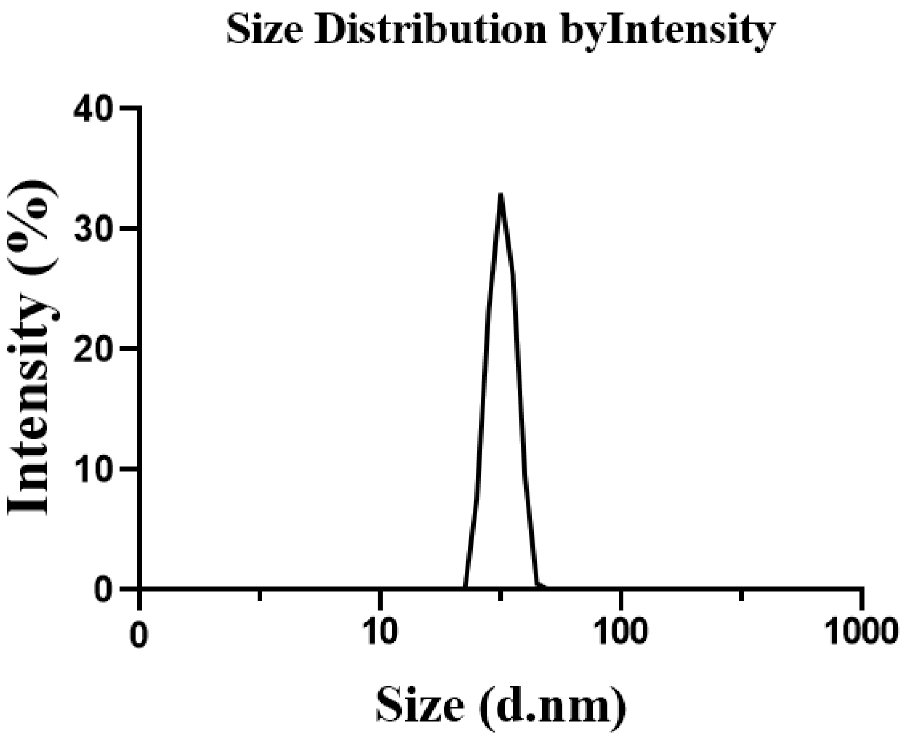

Figure S4. Particle size distribution of 7% ME was measured by DLS at 25°C

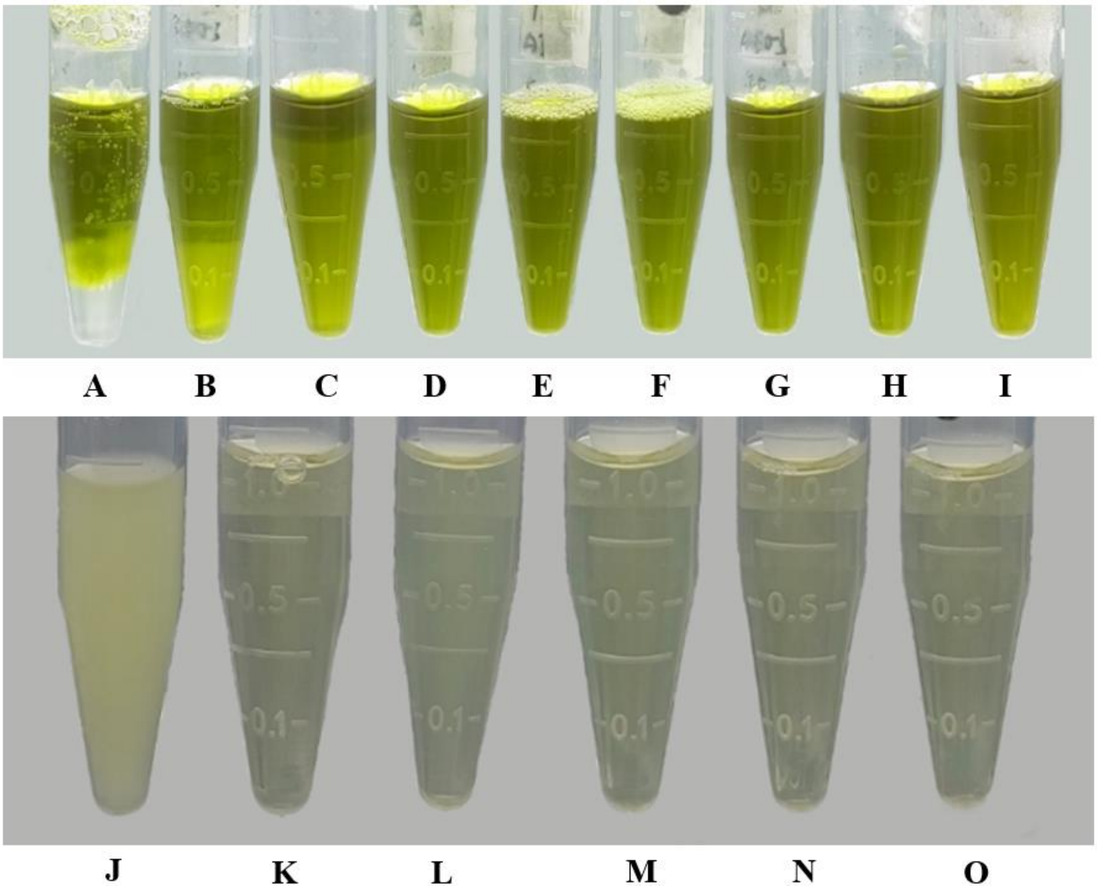

Figure S5. Visual assessment of CLE clarification in surfactants across a range of HLB values. Panels A–I illustrate the CLE's appearance prior to dilution (HLB 8 ~ 16), while Panels J–O present the results after water dilution (HLB 11 ~ 16).

Table S1. Impact of extraction parameters on antifungal activity and yield of CLE

The solvents and drying conditions		Inhibition rate (%)[c]		Extraction yield
		P. italicum	*P. digitatum*	
Thermally-dried[a]	100% Ethanol extract	44.1 ± 7.23 d[d]	100.00 ± 0.00 a	7.05 ± 0.08 a
	Petroleum ether extract	54.5 ± 3.92 bc	75.7 ± 1.15 b	3.25 ± 0.11 cd
	Ethyl acetate extract	49.1 ± 0.96 cd	100.0 ± 0.00 a	5.36 ± 0.35 b
air-dried[b]	100% Ethanol extract	53.9 ± 5.02 bc	100.0 ± 0.00 a	6.86 ± 0.74 a
	Petroleum ether extract	65.6 ± 3.49 a	100.0 ± 0.00 a	2.59 ± 0.71 d
	Ethyl acetate extract	60.1 ± 1.51 ab	100.0 ± 0.00 a	4.06 ± 0.24 c

[a] The CLE was dried in a hot drying oven (45 ± 1°C) until constant weight.

[b] The CLE was air-dried at room temperature (25 ± 1°C) until constant weight.

[c] The working concentration of all experimental extracts were standardized at 200.0 mg/L.

[d] different lowercase letters in the same column indicate significant differences among treatments ($p < 0.05$)

Table S2. Response surface methodology experimental design for factors

Level	Factor		
	A: Ultrasonic Temperature (°C)	B: Ultrasonic Time (h)	C: Solid to Liquid Ratio (m/v)
1	50	2	1:10
2	60	3	1:15
3	70	4	1:20

Table S3. Ethanol concentration modulates antifungal activity and extract yield of CLE

Treatment	Inhibition rate (%)		Extraction yield
	P. italicum	*P. digitatum*	
100% Ethanol extract	50.00 ± 0.00 a[a]	100.0 ± 0.00 a	7.32 ± 0.35 b
75% Ethanol extract	34.4 ± 0.00 b	100.0 ± 0.00 a	9.35 ± 0.47 a
50% Ethanol extract	31.3 ± 0.00 b	88.1 ± 0.00 b	7.77 ± 0.27 b

[a] different lowercase letters in the same column indicate significant differences among treatments ($p < 0.05$); identical lowercase letters indicate no significant difference ($p > 0.05$)

Table S4. Ultrasound temperature modulates antifungal activity and extract yield of CLE

Temperature (°C)	Inhibition rate (%)		Extraction yield
	P. italicum	*P. digitatum*	
20	17.8 ± 3.57 ab[a]	100.0 ± 0.00 a	5.3 ± 0.10 d
30	17.9 ± 3.57 ab	100.0 ± 0.00 a	6.53 ± 0.51 c
40	21.4 ± 0.00 ab	100.0 ± 0.00 a	7.63 ± 0.50 b
50	16.7 ± 2.06 b	100.0 ± 0.00 a	8.03 ± 0.71 b
60	23.8 ± 4.12 a	100.0 ± 0.00 a	9.00 ± 0.46 a
70	20.2 ± 5.45 ab	100.0 ± 0.00 a	8.60 ± 1.04 ab

[a] different lowercase letters in the same column indicate significant differences among treatments ($p < 0.05$); identical lowercase letters indicate no significant difference ($p > 0.05$)

Table S5. Antifungal activity and extract yield of CLE as a function of ultrasound treatment time

Time (h)	Inhibition rate (%)		Extraction yield
	P. italicum	*P. digitatum*	
0.5	17.9 ± 3.57 b[a]	100.0 ± 0.00 a	9.00 ± 0.46 d
1.0	23.8 ± 4.12 a	100.0 ± 0.00 a	9.73 ± 0.40 cd
1.5	23.8 ± 2.06 a	100.0 ± 0.00 a	10.6 ± 0.68 abc
2.0	22.6 ± 2.06 ab	100.0 ± 0.00 a	10.6 ± 0.23 abc
2.5	21.4 ± 3.57 ab	100.0 ± 0.00 a	11.0 ± 0.71 ab
3.0	22.6 ± 2.06 ab	100.0 ± 0.00 a	11.3 ± 0.15 a
4.0	23.1 ± 1.80 a	100.0 ± 0.00 a	10.3 ± 0.70 bc

[a] different lowercase letters in the same column indicate significant differences among treatments ($p < 0.05$); identical lowercase letters indicate no significant difference ($p > 0.05$)

Table S6. Antifungal activity and extract yield of CLE as a function of solvent-to-material ratio

Material-solvent ratio	Inhibition rate (%)		Extraction yield
	P. italicum	*P. digitatum*	
1:5	19.1 ± 2.06 a[a]	100.0 ± 0.00 a	6.27 ± 0.81 c
1:10	21.4 ± 3.57 a	100.0 ± 0.00 a	9.13 ± 1.29 b
1:15	22.6 ± 2.06 a	100.0 ± 0.00 a	11.3 ± 0.15 a
1:20	25.0 ± 3.57 a	100.0 ± 0.00 a	11.0 ± 0.45 b
1:25	22.6 ± 2.06 a	100.0 ± 0.00 a	8.00 ± 0.60 b

[a] different lowercase letters in the same column indicate significant differences among treatments ($p < 0.05$); identical lowercase letters indicate no significant difference ($p > 0.05$)

Table S7. Results of Box-Behnken experimental design

Group	A: Ultrasound temperature	B: Ultrasound time	C: Material-solvent ratio	Extraction yield
1	50	2	1:15	9.4
2	70	2	1:15	10.0
3	50	4	1:15	9.2
4	70	4	1:15	11.0
5	50	3	1:10	8.1
6	70	3	1:10	9.7
7	50	3	1:20	9.4
8	70	3	1:20	11.0
9	60	2	1:10	8.9
10	60	4	1:10	9.2
11	60	2	1:20	9.9
12	60	4	1:20	11.0
13	60	3	1:15	11.3
14	60	3	1:15	11.3
15	60	3	1:15	11.3
16	60	3	1:15	10.8
17	60	3	1:15	11.1

Table S8. Table of significance test for regression coefficients

Source of Variation	Sum of Squares	Degrees of Freedom	Mean Square	F-Value	*p*-Value	Significance
Model	16.12	9	1.79	38.9	< 0.0001	**
A	3.92	1	3.92	85.2	< 0.0001	**
B	0.61	1	0.61	13.2	0.0083	**
C	3.65	1	3.65	79.2	< 0.0001	**
AB	0.36	1	0.36	7.83	0.0266	*
AC	0.00	1	0.00	0.00	1.0000	
BC	0.16	1	0.16	3.46	0.1044	
A^2	2.24	1	2.24	48.8	0.0002	**
B^2	1.18	1	1.18	25.7	0.0014	**
C^2	3.26	1	3.26	70.9	0.0001	**
Residuals	0.32	7	0.046	-	-	
Lack of fit	0.13	3	0.043	0.90	0.5140	
Error	0.19	4	0.045	-	-	-
Sum	16.44	16	-	-	-	-

Table S9. Toxicity of the different compounds against *P. italicum* and *P. digitatum*

Penicillium Species	Compound	Toxicity regression equation	EC_{50}[a]（CI_{95}[b]）(mg/L)	r[c]
P. italicum	CLE	$y = 0.2765\log_{10}(x) - 0.1697$	264.4（182.9 ~ 420.9）	0.9458
	Thiabendazole	$y = 0.7436\log_{10}(x) - 0.6649$	36.9（28.6 ~ 45.9）	0.9801
P. digitatum	CLE	$y = 4.2536\log10(x) - 6.1744$	37.1（35.3 ~ 38.7）	0.9792
	Thiabendazole	$y = 1.0788\log10(x) + 1.6603$	0.08（0.05 ~ 0.11）	0.9301

[a] Effective dose for 50% inhibition compared with the control.

[b] 95% confidence intervals.

[c] Correlation coefficient.

Table S10. Toxicity of the 2,4-DTBP against *P. italicum* and *P. digitatum*

Compound	*Penicillium* Species	Toxicity regression equation	EC_{50}[a](CI_{95}[b])(mg/L)	r[c]
2,4-DTBP	*P. italicum*	$y = 0.5006\log(x) - 0.0562$	12.9 (8.6 ~ 18.1)	0.9505
	P. digitatum	$y = 0.4787\log(x) + 0.1115$	6.5 (3.3 ~ 10.0)	0.9311

[a] Effective dose for 50% inhibition compared with the control.

[b] 95% confidence intervals.

[c] Correlation coefficient.

Table S11. Solvent screening results

Solvent	Solubility at room temperature (30 ± 2 °C)	Stored at 4 °C for 3 days
Methanol	+++	No precipitate or layers formed
Ethanol	+++	No precipitate or layers formed
Isopropyl alcohol	++	Partial precipitate or layers formed
Ethyl acetate	+	/
Acetonitrile	+	/
1-Butanol	++	Partial precipitate or layers formed
Ethylene glycol	++	Partial precipitate or layers formed

Table S12. The clarifying effect of CLE in emulsifiers with different HLB values

HLB values	Phenomenon	Phenomenon After Dilution with Water
8	stratification	/
9	stratification	/
10	stratification	/
11	clarification	turbidity
12	clarification	clarification
13	clarification	clarification
14	clarification	clarification
15	clarification	clarification
16	clarification	clarification

Table S13. Binary surfactant combinations with HLB values greater than 12

Anionic surfactant	Nonionic surfactant	2:8 (AS:NS) HLB value range	3:7 (AS:NS) HLB value range
CDBS	1601#	14.9 ~ 15.3	13.9 ~ 14.5
	1602#	14.2 ~ 14.6	13.3 ~ 13.9
	T-20	14.6 ~ 15.0	13.7 ~ 14.3
	T-80	13.4 ~ 13.8	12.6 ~ 13.2

Table S14. Screening results of binary surfactants

Surfactant	Other Components	State	Dilute with water	In the refrigerator
CDBS (3%) + 1601# (7%)<br>CDBS (3%) + 1602# (7%)<br>CDBS (3%) + T-20 (7%)<br>CDBS (3%) + T-80 (7%)	CLE (7%) + Ethanol (32.8%) + Ethylene glycol (17.2%) + H_2O (30%)	Clarification	Significant precipitation formed	Precipitation formed after 3 d
CDBS (6%) + 1601# (24%)<br>CDBS (6%) + 1602# (24%)<br>CDBS (6%) + T-20 (24%)<br>CDBS (6%) + T-80 (24%)	CLE (7%) + Ethanol (40%) + H_2O (20%)	Clarification	Minimal precipitation formed	Minimal precipitation formed after 7 d

Table S15. Inhibitory rate of 30% binary surfactant-based microemulsions against *P. italicum* and *P. digitatum* at 200 mg/L

30% binary surfactant (2:8)		Inhibitory rate (%)	
		P. italicum	*P. digitatum*
CDBS	1601#	54.8 ± 4.12 b[a]	100 ± 0.00 a
CDBS	1602#	59.5 ± 4.13 b	100 ± 0.00 a
CDBS	T-20	67.9 ± 3.57 a	100 ± 0.00 a
CDBS	T-80	51.2 ± 2.06 b	100 ± 0.00 a

[a] different lowercase letters in the same column indicate significant differences among treatments ($p < 0.05$); identical lowercase letters indicate no significant difference ($p > 0.05$)

Table S16. Inhibitory activity of 7% ME against *P. italicum* and *P. digitatum* at 200 mg/L before and after heat storage

Sample	Inhibitory rate (%)	
	P. italicum	*P. digitatum*
7% ME	57.6 ± 5.61 a[a]	100 ± 0.00 a
7% ME (after heat storage)	59.3 ± 2.01 a	100 ± 0.00 a

[a] different lowercase letters in the same column indicate significant differences among

treatments ($p < 0.05$); identical lowercase letters indicate no significant difference ($p > 0.05$)

Table S17. Toxicity of 7% ME against *P. italicum* and *P. digitatum*

Treatment	*Penicillium* Species	Toxicity regression equation	EC_{50}[a]（CI_{95}[b]）（mg/L）	r[c]
7% ME	*P. italicum*	$y = 0.3503\log10(x) - 0.1447$	69.3 (58.3 ~ 83.1)	0.9690
	P. digitatum	$y = 1.7414\log10(x) - 1.9390$	25.2 (19.0 ~ 31.3)	0.9898
Blank ME	*P. italicum*	$y = 0.5182\log10(x) - 1.3778$	4204.5 (3006.1 ~ 5695.0)	0.9383
	P. digitatum	$y = 0.6212\log10(x) - 1.5947$	2356.0 (1816.8 ~ 3086.9)	0.9140

[a] Effective dose for 50% inhibition compared with the control.

[b] 95% confidence intervals.

[c] Correlation coefficient.

Table S18. *In vivo* control efficiency of 7% ME against green mold and blue mold

Treatment	Blue mold		Green mold	
	Disease index (%)	Control effect (%)	Disease index (%)	Control effect (%)
ddH_2O	100.0 ± 0.00 a[a]	-	78.3 ± 8.50 a	-
CLE	56.3 ± 7.77 b	43.7 ± 7.77 b	38.7 ± 6.11 b	50.6 ± 7.87 c
7% ME	22.6 ± 6.43 c	77.4 ± 6.43 a	4.30 ± 3.16 d	94.5 ± 4.04 a
Thiabendazole	29.0 ± 5.56 c	71.0 ± 5.56 a	14.7 ± 6.43 c	81.3 ± 8.23 b

[a] different lowercase letters in the same column indicate significant differences among treatments ($p < 0.05$); identical lowercase letters indicate no significant difference ($p > 0.05$)

Table S19.·Molecular docking analysis of 2,4-DTBP with CYP51 proteins of *P. italicum* (Q12664) and *P. digitatum* (A1XG20)

CYP51	Binding energy (kcal/mol)	Number of hydrogen bonds	Hydrogen bonding residues
Q12664	–7.39	3	Pro451, Ile370, and Ser368
A1XG20	–5.47	2	Ile371 and Pro452

Table S20. Primers used for qRT-PCR analysis in *P. italicum*

Primers	Sequence
β-Actin	TTACCTGATGAAGATCCTCGC(F)<br>GGGAGGATTTTATGTAGGCGT(R)
CYP51A	GTCTACGACTGTCCCAACTC(F)<br>GGCAAGAACGACTTCCTGAT(R)
CYP51B	TTTGTCGGAAGCACCATCAG(F)<br>AGACCTCTTCGGCGTTCACAT(R)
CYP51C	ATCTACCCACACATCCTCCTC(F)<br>CTGCTGTGTATGGCTGTGAG(R)
ERG6	TATGCTACCTTGACCCGACAC(F)<br>TAGTGCTCGTGGCGGGCAATAG(R)

Table S21. Primers used for qRT-PCR analysis in *P. digitatum*

Primers	Sequence
β-Actin	TCCACTACTGCCGAGCGTGAAAT(F)<br>CCGCCAGACTCAAGACCAAGAAC(R)
CYP51A	TGCTGGATTCTCCTTCGACT(F)<br>GCCCAAGTTGTTGACCTGTT(R)
CYP51B	CACCCAAAGTCGTGCAAAGTAT(F)<br>TTGACAAACTTCTTCTGCTCCA(R)
CYP51C	TGAGAAGCTCCAGAAATTGATT(F)<br>AAGCGACCTCATGAAGGGAAGA(R)
ERG6	CGCGTGATGCCGCCTTCAAC(F)<br>TGAGCCTTGCGGGCCTCACG(R)
